# The Immune Paradox in Acute Anorexia Nervosa: A Hypothesis Linking Hyperactivity, Restrained Inflammation, Preserved Innate Defense, and Constrained Adaptive Immunity

**DOI:** 10.3390/jcm15218538

**Published:** 2026-11-02

**Authors:** Lucille Lakier Smith

**Affiliations:** Human Performance Laboratory, Department of Kinesiology, College of Health & Human Performance, East Carolina University, Greenville, NC 27858, USA; lclakier@gmail.com

**Keywords:** acute anorexia nervosa (AN-acute), hyperactivity (HyAc), non-exercise activity thermogenesis (NEAT), immunometabolism, leptin, innate immunity, adaptive immunity, inflammation, myokine interleukin-6

## Abstract

**Background:** Anorexia nervosa (AN) is a severe psycho-metabolic disorder characterized by persistent energy restriction and substantial loss of adipose tissue. Despite profound undernutrition and measurable immune alterations, individuals with acute AN (AN-acute) have been reported to experience fewer clinically evident infections than expected from conventional models of malnutrition. This creates an apparent immune paradox. **Scope of Review:** Immune findings in AN are heterogeneous, reflecting differences in illness duration, eating-disorder subtype, nutritional severity, and methodology. Furthermore, persistent physical activity, a prominent feature of AN-acute, has received relatively little attention despite its important immunomodulatory effects in healthy individuals. This review presents a hypothesis integrating concepts from exercise physiology and immunometabolism with findings on immune function in AN-acute. **Hypothesis:** The immune phenotype of AN-acute may reflect selective allocation of limited energy, consistent with immunometabolic principles. It is proposed that severe energy restriction may permit sufficient neutrophil- and macrophage-mediated innate defense to contain pathogens early while constraining the more metabolically demanding activation and expansion of adaptive immunity. At the same time, multiple metabolic and endocrine mechanisms may restrain systemic inflammation, potentially reinforced by repeated contraction-derived IL-6 myokine signaling associated with persistent hyperactivity. **Conclusions:** This model proposes that preserved innate containment, constrained adaptive escalation, and restrained systemic inflammation interact in AN-acute to reduce the clinical expression of infection. It offers a testable framework linking starvation, hyperactivity, immunometabolism, and immune function.

## 1. Introduction

Anorexia nervosa (AN) is a psychiatric disorder that disproportionately affects females [1,2]. It is characterized by persistent restriction of energy intake despite food availability, resulting in substantial weight loss, often approaching 25%, driven primarily by depletion of adipose tissue with accompanying reductions in lean mass [3,4]. Comparable weight loss has also occurred during controlled semi-starvation [5]. 

A striking feature of AN-acute is the low incidence of clinically apparent infection despite severe emaciation. Individuals with AN reportedly experience fewer symptomatic viral infections than expected [6,7,8] whereas severe starvation and malnutrition in other contexts are often associated with increased infectious morbidity and mortality [9,10]. However, this comparison requires caution. Many reports of infection during starvation [9,10] or severe malnutrition involve prolonged or complex nutritional deprivation accompanied by protein and micronutrient deficiencies, greater pathogen exposure, poor sanitation, and other environmental or physiological stressors [10]. These factors make it difficult to isolate the effects of caloric restriction or weight loss on infectious burden. Severe malnutrition is therefore an informative comparison, but not a direct physiological equivalent of AN-acute.

Nevertheless, reports spanning several decades describe fewer clinically apparent infections than anticipated in AN, despite documented immune abnormalities or “dysregulation” [7,11,12]. This discrepancy, immune alterations coexisting with a low burden of overt infection, constitutes the AN-acute infection paradox.

Research on immune function in AN has produced heterogeneous and often inconclusive findings, reflecting differences in illness duration, nutritional severity, metabolic status, and methodology [7,13]. Another underexplored factor is persistent physical activity in AN-acute, which may influence immune function through exercise’s well-established immunomodulatory effects on healthy individuals [14,15]. 

This review proposes that immune alterations in AN-acute may reflect, at least in part, a functional adaptation to severe energy restriction rather than simply generalized immune dysfunction. The underlying premise is that, under profound energy limitations, physiological systems may favor less energy-intensive processes while still maintaining sufficient host defense.

For this hypothesis, AN-acute pragmatically refers to substantially underweight individuals with an illness duration of less than three years, consistent with prior staging work that defines an early-stage group as illness duration < 3 years [2].

Three features of AN-acute are particularly relevant to the present model:

1. Low body weight: BMI is markedly reduced, falling below 15 kg/m^2^ in severe cases and nearer 17 kg/m^2^ in milder presentations [16].
2. Reduced energy intake: Dietary energy intake is markedly reduced in AN compared with healthy controls, although absolute intake and macronutrient composition vary across studies and according to assessment method [17,18,19].
3. Hyperactivity/compulsive movement: Casper et al. [20] found that 95.1% of adolescent females with restricting-type AN reported increased movement drive or restlessness, consistent with historical and contemporary observations [21,22,23,24]. In this hypothesis, HyAc is a fundamental feature of AN-acute and a starvation-associated motor activation pattern with potential effects on multiple physiological systems, including immunity [25,26,27,28].

The purpose of this review is to integrate evidence from immunology, immunometabolism, starvation physiology, and exercise physiology to examine how the differing energy demands of immune processes may shape immune function in AN-acute. It proposes that adaptations to severe caloric restriction, together with persistent HyAc, may selectively preserve some immune functions, constrain others, and restrain systemic inflammation, thereby contributing to the limited clinical expression of infection.

## 2. Proposed Hypothesis

The central premise is that energy allocation principles may better explain the infection paradox in AN-acute. Immune responses are metabolically demanding, requiring immune cell activation, cytokine production, lymphocyte proliferation, antibody synthesis, and tissue repair [29,30]. During prolonged starvation, increased movement compounds these demands, and they compete for energy needed to maintain vital physiological functions.

Life History Theory posits that when energy is limited, organisms allocate resources among competing biological functions to favor immediate survival [31,32]. In this context, energy-efficient immunity refers to preferentially using immune mechanisms that provide meaningful host protection while minimizing metabolic expenditure.

The hypothesis has three components:

1. Innate immune functions may remain relatively preserved under severe energy restriction and may be further influenced by persistent HyAc, allowing early pathogen recognition and containment despite reductions in cell number or selected functions.
2. Adaptive immune activation and clonal expansion may be more strongly constrained because T- and B-cell activation, differentiation, and proliferation require substantial nutrient and biosynthetic investment.
3. An inflammation-restrained systemic environment may arise from starvation physiology associated with persistent HyAc, which may be an important modifier. Repeated physical activity generates autonomic, endocrine, metabolic, thermal, and myokine signals that influence immune function.

Taken together, the model proposes a selective reorganization of host defense under severe energy restriction and persistent HyAc rather than generalized immune failure. This framework is broadly consistent with immune findings reported in AN-acute [7,8] but remains hypothetical and requires direct experimental testing.

The review develops this model by outlining immune organization and immunometabolism, then examining key metabolic regulators, systemic inflammatory signals, innate and adaptive immune function, and the potential modifying role of HyAc in AN-acute.

## 3. Hyperactivity in Acute Anorexia Nervosa

Persistent HyAc is one of the most characteristic and enduring clinical features of AN-acute. Since Gull’s original description in 1874 [21] researchers have consistently documented excessive physical activity across patient populations and treatment settings [33,34]. Although often viewed as a secondary behavioral symptom, the present review proposes that HyAc may be central to AN-acute physiology and may exert meaningful immunological effects.

This perspective matters because regular moderate exercise strongly modulates immune and inflammatory function in healthy individuals [35,36]. Understanding the amount, type, and physiological impact of physical activity in AN-acute is therefore essential for evaluating its proposed contribution to the immune phenotype.

### 3.1. Components of Hyperactivity

In this review, HyAc refers to three related forms of movement that together contribute to total daily activity and energy expenditure [28] and potentially immune function:

- Excessive Exercise (ExEx). Structured, intentional physical activity such as running, cycling, swimming, resistance training, yoga, or organized sports. Intensity and duration vary widely.
- Non-Exercise Activity Thermogenesis (NEAT). Energy expended during routine daily activities outside formal exercise, including walking, shopping, household chores, occupational tasks, standing, and pacing [37]. Because these activities occur repeatedly throughout the day, NEAT can substantially increase total daily activity.
- Spontaneous Physical Activity (SPA). Low-intensity, non-goal-directed movements such as fidgeting, postural adjustments, pacing, and frequent sit-to-stand transitions. SPA is often grouped within NEAT but is distinguished here because these movements may arise spontaneously and reflect an illness-related motor drive rather than deliberate activity [38].

### 3.2. Physiological Significance of NEAT in AN-Acute

Although NEAT is conventionally distinguished from structured exercise, some forms may impose a meaningful physiological workload in AN-acute. In healthy controls HCs, increased workload activates the sympathetic nervous system, raising heart rate (HR) and reducing vagally mediated HRV. Individuals with AN-acute typically show low resting HR and higher parasympathetically mediated HRV [39,40]. Yet even mild activity can rapidly alter this pattern. Billeci et al. [41] found that light physical activity elicited marked changes in HR and HRV in adolescents with AN-acute, whereas controls showed minimal autonomic responses. Thus, activities that are physiologically trivial in HCs may represent a greater relative workload in AN-acute.

This is particularly relevant to prolonged walking, which many patients do not recognize as exercise. Kolnes [42] described individuals who walked several hours per day yet denied exercising. Assessments limited to structured ExEx may therefore underestimate the cumulative physiological impact of repeated low-intensity muscle contractions during NEAT.

A likely explanation for this exaggerated response is reduced aerobic capacity in AN-acute [43] making identical absolute workloads more demanding. Sustained walking and other whole-body NEAT may repeatedly activate sympathetic, endocrine, metabolic, and thermal pathways while increasing energy expenditure. The proposal is not that all NEAT is exercise-like, but that repeated or prolonged whole-body activity may become physiologically significant under severe energy restriction.

### 3.3. Implications of Hyperactivity for the Present Immune Hypothesis

This broader view of HyAc is central to the proposed immune model. In healthy individuals, regular moderate exercise influences immune cell trafficking, innate and adaptive immune function, and inflammatory regulation [15,35]. Persistent HyAc in AN-acute, including physiologically meaningful NEAT, may therefore provide recurring sympathetic, endocrine, metabolic, thermal, and myokine signals that shape immune function.

These effects are particularly relevant to mechanisms discussed later, including muscle-derived IL-6, cortisol, neutrophil mobilization, immunometabolic signaling, and activity-induced warming. If confirmed, assessments limited to structured exercise may underestimate both the physiological burden of HyAc and its contribution to the AN-acute immune phenotype.

Experimental evidence relevant to several of these mechanisms comes from the activity-based anorexia (ABA) model. First described in the late 1960s, ABA combines restricted food access with voluntary running-wheel activity, producing progressive weight loss and marked hyperactivity. The model does not fully capture the psychological and clinical complexity of human AN-acute, but it reproduces several relevant physiological features and has been widely used to study starvation-associated hyperactivity, neuroendocrine and metabolic adaptations, stress and reward pathways, and thermoregulation. The relevant sections below consider findings from the ABA model that parallel or help explain the physiological features of human AN-acute [44].

## 4. The Immune System

### 4.1. Evolutionary Organization of the Immune System

The immune system comprises two highly integrated branches: innate and adaptive immunity. Innate immunity is evolutionarily ancient and provides the immediate foundation of host defense, whereas vertebrate adaptive immunity emerged approximately 500 million years ago, adding highly specific antigen recognition and immunological memory. Although the two systems differ in evolutionary origin, speed, specificity, and mechanisms of activation, they function cooperatively, with innate immune signals helping to initiate and shape adaptive responses [45,46]. These evolutionary and metabolic differences provide important background for the hypothesis proposed in this review.

### 4.2. Functional Organization of the Immune System

Innate immunity provides the body’s immediate defense against invading pathogens by recognizing conserved microbial or damage signals and rapidly initiating antimicrobial activity, inflammation, clearance, and repair [47]. Innate responses are not inherently pro-inflammatory. Depending on the physiological context, they may promote inflammatory defense, metabolic regulation, resolution, or tissue repair.

Adaptive immunity provides highly specific protection through antigen-specific T and B lymphocytes and generates long-lasting immunological memory [48]. During a novel infection, rare antigen-specific lymphocytes must first activate, then undergo extensive clonal expansion and differentiation before a substantial adaptive response develops [49]. 

Dendritic cells (DCs) link the two systems: they capture microbial antigens during innate surveillance and, after appropriate activation, initiate adaptive immunity by activating naïve T cells [50]. This DC–T-cell transition is central to the present hypothesis because metabolic constraint here could limit adaptive upregulation while leaving substantial innate function available.

### 4.3. Energetics of Innate and Adaptive Immunity

Both innate and adaptive immune cells increase their metabolic activity during activation, making immune function sensitive to nutrient and energy availability [29,30]. The important distinction is not that innate immunity is metabolically inexpensive, but that many innate effector cells are already present and can respond rapidly, whereas a primary adaptive response requires activation and extensive clonal expansion of antigen-specific lymphocytes.

Full adaptive upregulation therefore requires sustained nutrient uptake, biosynthesis, cellular growth, and repeated cell division. Under severe, prolonged energy restriction, this additional metabolic investment may make adaptive expansion particularly vulnerable to constraints.

This leads to the model’s central proposal: in AN-acute, limited resources may permit sufficient rapid innate defense while restricting progression to the greater metabolic investment required for adaptive expansion. Later sections examine the specific roles of neutrophils, macrophages, and dendritic cells in maintaining early innate defense, as well as potential metabolic constraints at the DC–T-cell transition.

## 5. Immunometabolism During Energy Restriction

**Figure 1:**
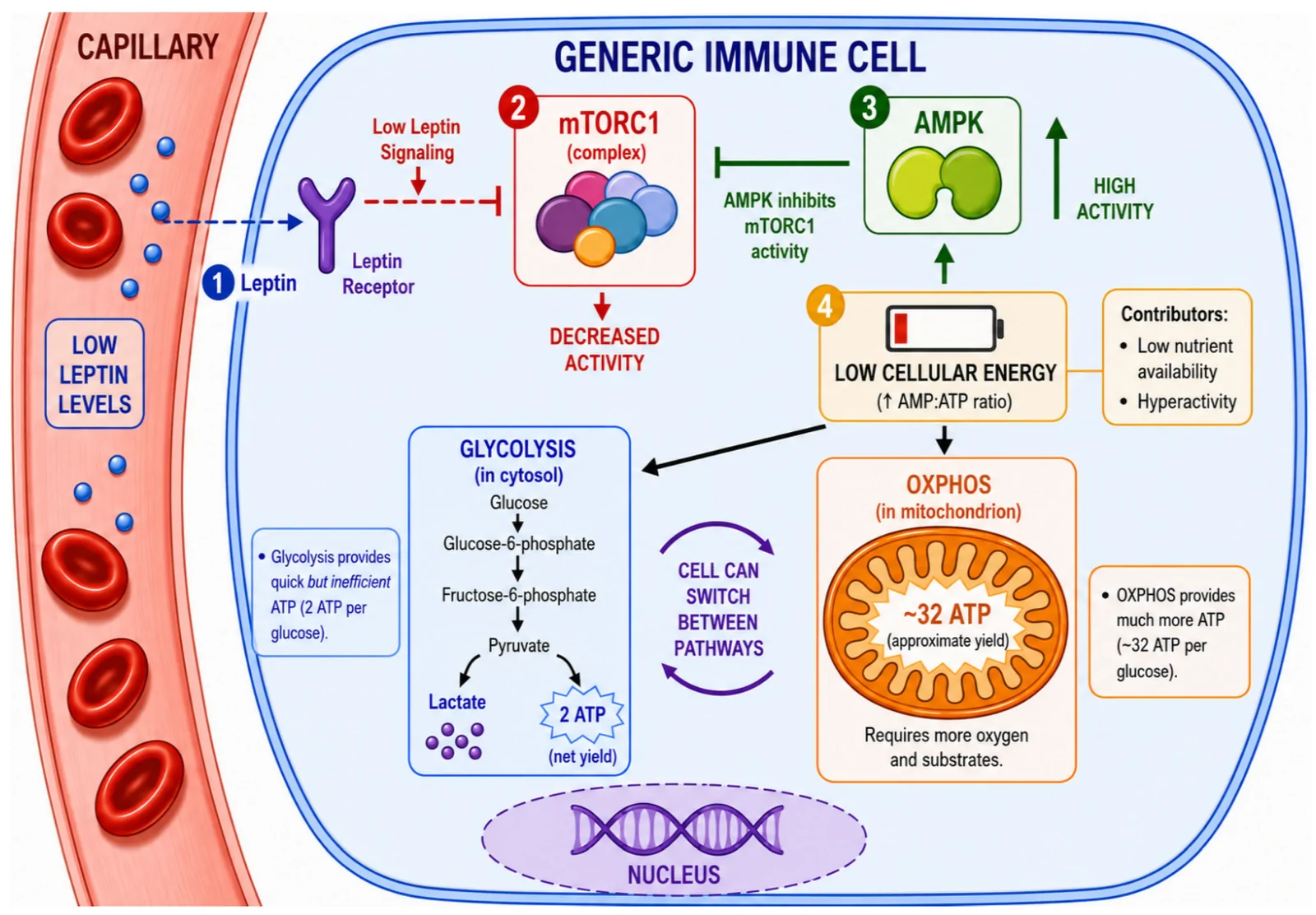
Metabolic regulation of immune-cell activation during severe energy restriction. Low circulating leptin reduces leptin receptor signaling, thereby limiting mTORC1 activation, while reduced cellular ATP availability activates AMP-activated protein kinase (AMPK), which further suppresses mTORC1 activity. Together, these signals favor an energy-conserving cellular state rather than the anabolic metabolic reprogramming required for full immune-cell activation and proliferation. Immune cells can generate ATP through glycolysis, which provides rapid but inefficient ATP production, or through mitochondrial oxidative phosphorylation (OXPHOS), which yields substantially more ATP but requires adequate oxygen and metabolic substrates. In acute anorexia nervosa, severe nutrient restriction, potentially compounded by persistent hyperactivity, may therefore shift the AMPK–mTORC1 balance toward energy conservation and constrain energetically demanding immune activation. Abbreviations: AMPK, AMP-activated protein kinase; ATP, adenosine triphosphate; mTORC1, mechanistic target of rapamycin complex 1; OXPHOS, oxidative phosphorylation.

Metabolism refers to the biochemical processes that convert nutrients into energy (e.g., ATP) and molecules required for cellular function. Immune cells use several metabolic pathways and adjust their fuel use according to nutrient availability and functional demands. Their ability to activate, proliferate, differentiate, produce cytokines, and perform effector functions is therefore shaped by both metabolic fuel availability and the capacity to activate the metabolic pathways required for each response. (Figure 1).

### 5.1. Immunometabolism: Metabolism as a Regulator of Immune Function

Immunometabolism examines how metabolism and energy availability regulate immune-cell function. Metabolic conditions influence whether immune cells remain quiescent, activate, proliferate, migrate, produce inflammatory mediators, repair tissue, or form long-term memory. Thus, metabolism provides not only fuel but also signals that govern immune-cell behavior [29,30].

This concept is particularly relevant to AN-acute, where prolonged energy restriction combined with persistent HyAc creates sustained metabolic stress and may strongly influence how immune cells allocate and use limited resources [51].

### 5.2. Energy Availability and Metabolic Fuels

Immune cells use substrates such as glucose, fatty acids, and ketones to produce ATP and provide the cellular building blocks required for immune function. The availability of these fuels can therefore influence both the magnitude and character of an immune response [29,30]. 

During prolonged energy restriction, immune cells must adjust their metabolism to balance immediate functional demands with cellular maintenance and survival. This metabolic flexibility may allow some functions to continue even when resources for extensive activation or proliferation are limited.

### 5.3. Major Metabolic Pathways

Immune cells use several metabolic pathways, with glycolysis and oxidative phosphorylation being particularly important [29,30]. Glycolysis rapidly converts glucose into ATP and metabolic intermediates that support rapid immune activation and biosynthesis. Many immune cells increase glycolysis when they need rapid functional responses, growth, or proliferation.

Oxidative phosphorylation (OXPHOS) occurs within mitochondria and generates ATP using energy derived from several substrates, including fatty acids, glucose-derived pyruvate, amino acids, and ketone bodies. However, under severe caloric restriction, OXPHOS relies primarily on fatty acids and ketone bodies, with amino acid oxidation contributing minimally to conserve protein. OXPHOS commonly supports cellular maintenance, sustained function, and many regulatory or memory states.

The relative importance of these pathways varies across immune cell types and functional states. Neutrophils, for example, rely heavily on glycolysis even at baseline, whereas lymphocytes and macrophages can reprogram their metabolism following activation.

### 5.4. Metabolic Reprogramming During Immune Activation

A major advance in immunology is the recognition that immune cells reprogram their metabolism to meet functional demands. Activation often increases nutrient uptake and glycolysis, whereas other functions may depend more strongly on mitochondrial metabolism, fatty-acid oxidation, or combinations of pathways [29,30]. These metabolic programs help shape inflammatory, regulatory, antimicrobial, and tissue-repair functions.

This principle is especially relevant to adaptive immune upregulation. A primary T- or B-cell response requires antigen-specific lymphocytes to activate, proliferate, differentiate, and undergo extensive clonal expansion. These processes require substantial increases in nutrient uptake, protein synthesis, lipid and nucleotide biosynthesis, and overall metabolic activity [30,52]. Prolonged energy deficiency may therefore be particularly important for adaptive expansion, not because innate immunity is metabolically inexpensive but because a substantial primary adaptive response requires generating large numbers of new antigen-specific cells.

### 5.5. Implications for AN-Acute

This framework underpins a central hypothesis of this review. How immune cells recognize and respond to this limited energy availability depends on several interacting metabolic signals. Three particularly relevant regulators are leptin, AMP-activated protein kinase (AMPK), and mechanistic target of rapamycin complex 1 (mTORC1).

## 6. Key Metabolic Regulators of Immune Function

### 6.1. Leptin: A Systemic Signal of Energy Availability

Leptin is an adipocyte-derived hormone with cytokine-like properties, and its circulating concentration broadly reflects body-fat stores. Individuals with AN-acute therefore develop profound hypoleptinemia as adipose tissue is depleted [53,54]. ABA models have shown similar reductions in leptin signaling [53]. 

Low leptin is an important systemic signal of energy deprivation. In the hypothalamus, falling leptin concentrations suppress energetically costly reproductive and thyroid functions and drive other neuroendocrine adaptations that conserve energy during prolonged starvation [55]. 

Leptin also links nutritional status to immune function. Adequate leptin signaling supports T-cell activation, proliferation, cytokine production, effector differentiation, and the metabolic reprogramming required to sustain these processes [56,57]. Experimental starvation reduces leptin and impairs T-cell responses, whereas leptin replacement can restore aspects of T-cell function [56]. Particularly relevant to the present hypothesis, leptin has been described as metabolically “licensing” activated T cells by supporting the increased glucose uptake and metabolic activity required for proliferation and effector function [57]. Leptin and the broader nutritional and metabolic environment also influence B-cell survival, proliferation, differentiation, and antibody production [58]. Thus, profound hypoleptinemia could affect both arms of adaptive immunity, not just T-cell responses.

Leptin also influences innate immune cells, including neutrophils, monocytes/macrophages, and dendritic cells. Profound hypoleptinemia should therefore not be viewed as selectively affecting adaptive immunity [58]. However, cells already present can perform many immediate innate antimicrobial functions without the extensive clonal proliferation characteristic of a primary adaptive response. Substantial adaptive upregulation, by contrast, requires antigen-specific lymphocytes to activate, proliferate, differentiate, and undergo extensive clonal expansion.

In AN-acute, very low leptin may therefore create a metabolic environment in which selected innate functions remain available, while full adaptive expansion becomes harder to initiate and sustain. This interpretation remains hypothetical, because leptin-dependent metabolic signaling in immune cells has not been directly characterized in AN-acute.

### 6.2. AMPK: The Intracellular Energy-Stress Sensor

AMP-activated protein kinase (AMPK) is a central intracellular energy sensor that is activated when cellular energy availability declines, as reflected by changes in the ATP/AMP/ADP balance. Once activated, AMPK acts as a metabolic brake on energy-intensive processes: it suppresses anabolic activities such as cell growth and protein synthesis while promoting ATP-generating pathways and cellular maintenance and survival [59,60]. 

Under conditions of adequate energy availability, AMPK activity need not constrain the metabolic reprogramming required for immune activation. However, when cellular energy becomes limited, increased AMPK signaling shifts resources away from growth and biosynthesis toward energy conservation. 

One key action of AMPK is inhibition of mTORC1, a nutrient-sensitive pathway that promotes cell growth and biosynthesis [61]. This is particularly relevant to lymphocyte activation. When T cells are activated, they require substantially more energy and nutrients to grow and proliferate. During nutrient limitation, AMPK helps maintain cellular energy balance and metabolic flexibility, but it can also constrain energy-intensive anabolic processes required for sustained proliferation [62]. Thus, AMPK is better viewed not simply as an inhibitor of immune activation, but as a *metabolic checkpoint* that helps match the scale of the immune response to available cellular energy.

A similar pattern may occur in AN-acute. Severe caloric restriction creates sustained systemic energy scarcity, while persistent HyAc further strains already limited fuel supplies. Together, starvation and continued activity could favor energy-stress signaling in immune cells, potentially increasing reliance on AMPK-dependent pathways that support cellular maintenance and metabolic adaptation while constraining metabolically costly processes such as sustained lymphocyte growth and clonal expansion.

Direct evidence for altered AMPK signaling in AN-acute is limited. In the ABA model, Fraga et al. [63] found no detectable changes in hypothalamic AMPK signaling despite profound metabolic effects, suggesting that hypothalamic AMPK may not mediate energy metabolism in key hypothalamic pathways. However, this finding is specific to the hypothalamus and does not exclude AMPK activation in peripheral immune cells, where AMPK responds directly to local cellular energy stress and nutrient deprivation [62].

Importantly, researchers have not directly demonstrated increased AMPK activity in immune cells in AN-acute. Its proposed role in this model therefore remains hypothetical. Nevertheless, AMPK provides a plausible mechanistic link between cellular energy status, mTORC1 signaling, and the metabolic requirements of immune activation.

### 6.3. Mechanistic Target of Rapamycin Complex 1 (mTORC1)

mTORC1 is a central nutrient- and growth-sensing pathway that integrates signals from amino acids, growth factors, hormones, and cellular energy availability. Functionally, it can be considered a growth permission signal. When nutrients and energy are sufficient and permissive hormonal signals are present, mTORC1 supports protein synthesis, cellular growth, activation, and proliferation. When resources are scarce, its activity declines [61].

This pathway is particularly important during T-cell activation, which requires a major increase in nutrient uptake and biosynthetic capacity [30]. Reduced mTORC1 signaling during nutrient deprivation can therefore constrain T-cell differentiation and proliferation. Importantly, experimental starvation and reduced leptin signaling have been shown to decrease mTOR activity and impair CD4+ effector T-cell proliferation [64].

AMPK and mTORC1 function as opposing but coordinated regulators of cellular resource use. When energy and nutrient availability are sufficient, mTORC1 supports anabolic growth and immune cell activation. During cellular energy stress, increased AMPK signaling can restrain mTORC1 and reduce energy-consuming biosynthesis [59,65].

In AN-acute, severe caloric restriction, reduced nutrient availability, profound hypoleptinemia, and persistent HyAc could collectively favor increased AMPK and reduced mTORC1 signaling. Such an environment may be particularly consequential for adaptive immunity, because activation and clonal expansion require sustained nutrient uptake, biosynthesis, and cell growth [30,60].

Despite these constraints, during low energy availability, relatively quiescent naïve and memory T and B cells may be maintained because they require substantially less energy for basic survival than for activation, differentiation, and extensive clonal expansion. Thus, the adaptive immune system may remain present but have a reduced capacity to mount a large new response. This interpretation is consistent with the continued presence of lymphocytes in AN-acute despite reported alterations in lymphocyte populations and activation characteristics [7,66].

Studies of immune-cell numbers and functional responses in AN-acute have, however, yielded heterogeneous findings, with some cells and functions remaining relatively preserved [7,66,67].

Moreover, researchers have not directly demonstrated altered AMPK–mTORC1 signaling in immune cells from individuals with AN-acute. The proposed AMPK↑–mTORC1↓ metabolic constraint should therefore be regarded as a biologically plausible and testable component of the present hypothesis rather than an established feature of AN.

## 7. Systemic Signals Shaping Inflammation and Fuel Allocation in AN-Acute

### 7.1. Cytokine Patterns and the Limits of Interpreting Inflammation in AN-Acute

Circulating cytokines provide valuable information about immune activity, but cytokine concentrations alone do not define the overall inflammatory state. Cytokine findings in AN-acute are heterogeneous. Meta-analytic evidence indicates that IL-6 is elevated in AN, while findings for other inflammatory cytokines are less consistent [68]. Thus, the available evidence does not support a uniformly pro-inflammatory state.

A key limitation is that researchers often measure cytokines from single resting or fasting blood samples without adequately accounting for recent physical activity. This is particularly relevant to IL-6, because skeletal muscle can release substantial amounts of myokine IL-6 during contraction [69].

### 7.2. IL-6 Source Matters: Immune-Derived Versus Muscle-Derived Signaling

Researchers frequently report elevated circulating IL-6 in AN-acute and often interpret it as evidence of inflammation. However, circulating IL-6 concentration alone cannot determine its biological function, as multiple sources produce IL-6. During infection or tissue injury, immune cells produce IL-6 as part of a broader inflammatory response, typically accompanied by cytokines such as IL-1β and TNF-α. In contrast, contracting skeletal muscle releases IL-6 into circulation, where it acts as a myokine with important metabolic and inflammation-limiting actions [69].

Different signaling pathways also shape IL-6’s functional profile. In classical signaling, IL-6 binds to membrane-bound IL-6 receptors on a restricted set of cells, including hepatocytes and select leukocyte subsets. This pathway generally supports metabolic regulation, regenerative responses, and short-lived anti-inflammatory effects. In pro-inflammatory trans-signaling, IL-6 binds to soluble IL-6 receptors in the circulation, enabling IL-6 to act on a much broader range of cells. This pathway promotes leukocyte recruitment and sustained inflammatory activity and is considered the pro-inflammatory arm of IL-6 biology [70] (Table 1).

Exercise-induced IL-6 can increase markedly, particularly during prolonged activity; increases approaching 100-fold have been reported after strenuous endurance exercise, although smaller increases are more common [69]. Persistent HyAc in AN-acute, including prolonged walking and repeated bouts of activity, therefore provides a plausible recurring stimulus for skeletal-muscle IL-6 release.

This hypothesis proposes that a substantial, potentially predominant, component of elevated circulating IL-6 in AN-acute may be muscle-derived rather than a marker of conventional systemic inflammation. Persistent HyAc could repeatedly generate the myokine IL-6, whose physiological effects favor metabolic regulation and limit inflammatory amplification. This model also plausibly involves a relative predominance of classical anti-inflammatory signaling over pro-inflammatory trans-signaling. However, neither the tissue source of circulating IL-6 nor the balance between these signaling pathways has been directly established in AN-acute.

### 7.3. Myokine IL-6 as a Link Between Hyperactivity, Fuel Mobilization, and Inflammation Restraint

Muscle-derived IL-6 may perform two particularly relevant functions.

**Figure 2:**
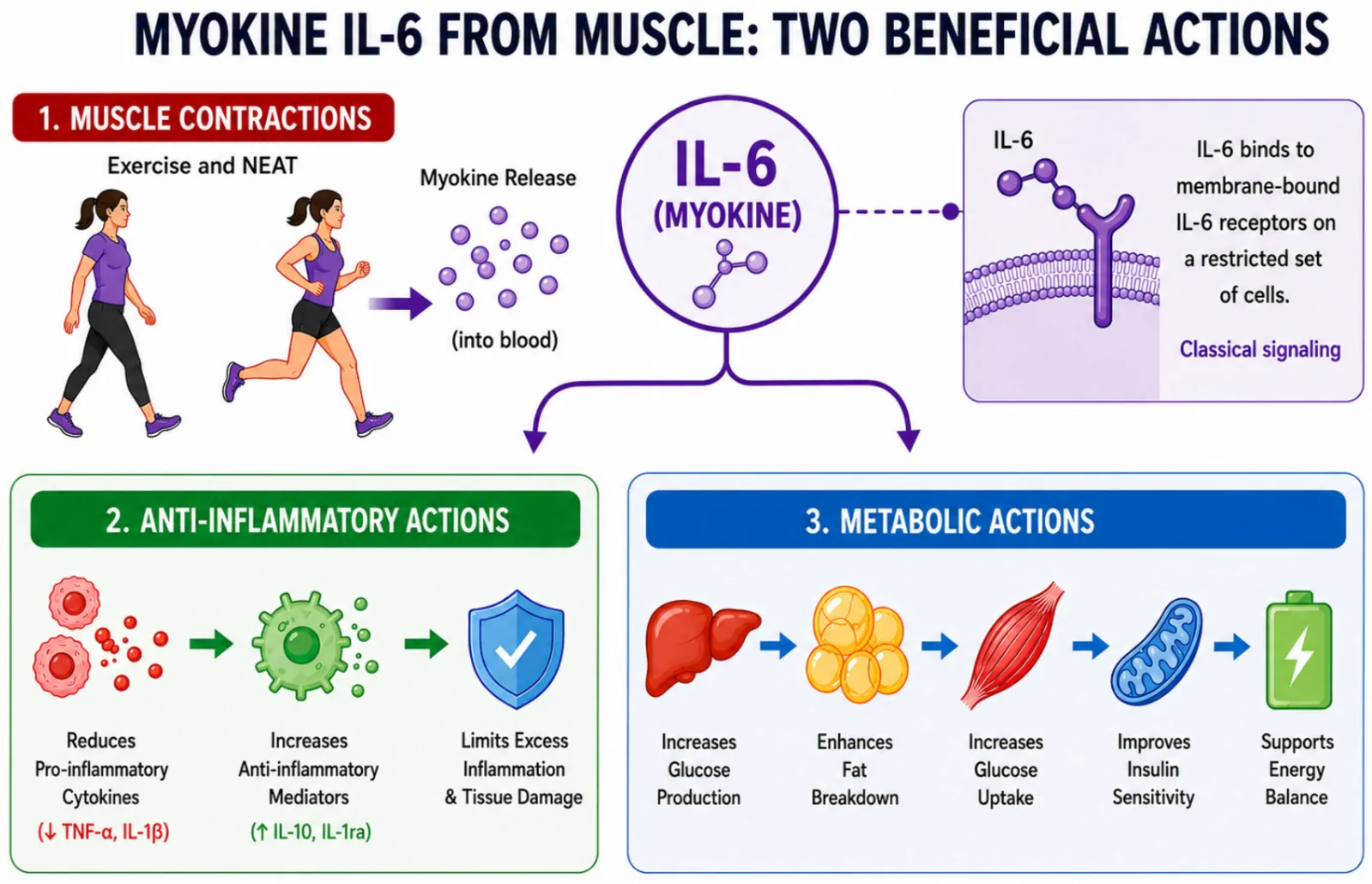
Muscle-derived IL-6 released during contraction may exert coordinated anti-inflammatory and metabolic effects.

- First, it contributes to the exercise-induced anti-inflammatory response by increasing mediators such as IL-1 receptor antagonist and IL-10, and by limiting TNF-α production [69].
- Second, it helps coordinate fuel availability during muscular activity by influencing glucose uptake and glucose homeostasis and promoting lipolysis and fatty-acid oxidation [69,72] (Figure 2).

In AN-acute, repeated IL-6 myokine signaling could therefore help mobilize scarce metabolic fuels while restraining unnecessary inflammatory amplification. This provides a potential mechanistic link between persistent HyAc, metabolic adaptation, and the inflammation-restrained systemic phenotype proposed in this review.

Future studies should determine the source and signaling characteristics of circulating IL-6 in AN-acute and relate these to recent physical activity, additional inflammatory markers, and metabolic responses. Measurements before and after both structured exercise and prolonged low-intensity activity would be especially informative. In addition, data on IL-6 in the ABA model are limited, and no direct evidence supports increased myokine IL-6 in ABA.

Whereas myokine IL-6 may help coordinate fuel use during continued activity, cortisol may complement these adaptations by mobilizing stored substrates while also restraining inflammatory signaling.

### 7.4. Hypercortisolemia: Inflammation Restraint and Fuel Mobilization

Hypercortisolemia is one of the most consistent endocrine abnormalities in AN-acute. A systematic review and meta-analysis of 101 studies involving more than 2500 females found significantly higher cortisol concentrations in women with AN-acute than in normal-weight controls [73]. Severe energy deficiency activates the hypothalamic–pituitary–adrenal axis, and persistent HyAc may serve as an additional physiological stressor [74]. The ABA model also reproduces the HPA-axis activation observed in human AN-acute and shows significantly elevated circulating corticosterone, the principal rodent glucocorticoid, paralleling the hypercortisolemia reported in patients with AN [75].

Cortisol has two broad actions that are central to the present hypothesis. First, it regulates immune activity by reducing inflammation and limiting adaptive immune activation. It suppresses pro-inflammatory cytokine signaling, including IL-1β, TNF-α, and IL-6, and restrains lymphocyte activation, differentiation, and proliferation [76].

Cortisol also reshapes innate immune activity. It increases circulating neutrophil numbers by redistributing and releasing them from the bone marrow, while reducing their movement from the bloodstream into tissues. Thus, higher blood neutrophil counts do not necessarily indicate greater participation in local inflammation. Cortisol also reduces pro-inflammatory cytokine production by monocytes and macrophages and can favor macrophage functions associated with inflammation resolution and tissue maintenance (M2-like). It inhibits dendritic cell maturation and antigen presentation, further limiting the initiation of adaptive immune responses [76].

In this way, glucocorticoids do not simply “switch off” immunity; they redistribute and restrain innate and adaptive immune activity to prevent excessive or energetically costly inflammatory responses [76].

Second, cortisol plays a central role in metabolism by maintaining the body’s energy supply during starvation and physiological stress. It helps the liver generate new glucose and mobilizes stored fuels by breaking down muscle protein and releasing fatty acids from adipose tissue. These processes increase the availability of amino acids, glycerol, and fatty acids, which the body converts into energy when food intake is extremely limited. Cortisol also reduces glucose utilization in less critical tissues, thereby preserving circulating glucose for organs with immediate metabolic needs, such as the brain [77,78].

Within the present hypothesis, hypercortisolemia in AN-acute may therefore serve a dual function: restraining energetically costly immune-inflammatory activity while maintaining access to metabolic fuel during severe energy scarcity. By limiting adaptive immune activation, modulating innate immunity, and mobilizing internal energy stores, cortisol may help preserve resources for essential physiological functions.

### 7.5. Elevated Adiponectin: Fuel Efficiency, Inflammation Restraint, and Macrophage Regulation

Adiponectin is an adipocyte-derived hormone with important metabolic and immune-regulatory functions. Unlike leptin, which declines markedly with adipose tissue loss, circulating adiponectin is paradoxically elevated in AN-acute. Pannacciulli et al. [79] reported significantly higher adiponectin concentrations in women with AN-acute.

Adiponectin may be particularly relevant during prolonged energy restriction because it promotes fatty acid oxidation, enhances insulin sensitivity, and supports metabolic fuel utilization in active tissues [80,81]. These actions may help optimize the use of limited metabolic substrates.

Adiponectin also has important inflammation-limiting effects. It can reduce macrophage production of pro-inflammatory mediators such as TNF-α and IL-6, increase anti-inflammatory mediators including IL-10, and promote macrophage characteristics associated with tissue repair and resolution of inflammation [82].

Within the present hypothesis, elevated adiponectin in AN-acute may therefore contribute to more efficient fuel utilization and restraint of excessive inflammatory activity, including modulation of macrophage function. Its elevation is therefore consistent with, rather than proof of, the proposed inflammation-limiting immunometabolic state of AN-acute.

### 7.6. Ketone Bodies: Alternative Fuel and Selective Inflammatory Restraint

Ketone bodies rise during prolonged caloric restriction as the body shifts from greater reliance on glucose toward increased use of stored fat. The principal circulating ketone, β-hydroxybutyrate (BHB), functions both as an alternative fuel during starvation and as a metabolic signaling molecule. Ketone production also increases during fasting and prolonged exercise, particularly when carbohydrate availability is limited [83].

BHB suppresses specific innate inflammatory pathways. Most notably, it inhibits an inflammasome, a danger-sensing complex in macrophages and other innate immune cells that normally promote production of the pro-inflammatory cytokines IL-1β and IL-18 [84]. BHB may therefore reduce inflammatory amplification.

Ketone bodies can also be elevated in individuals with AN-acute during severe caloric restriction [85]. Persistent HyAc could potentially further increase ketone production and utilization under conditions of limited carbohydrate availability [86].

Within the present hypothesis, increased ketone availability could therefore have two complementary effects: providing an alternative metabolic fuel when glucose is scarce and selectively restraining excessive inflammatory signaling [83,84]. BHB may therefore contribute to the proposed inflammation-limiting immunometabolic state of AN-acute, although its specific role in AN-acute and the extent to which HyAc modifies these effects remain hypothetical.

## 8. Cellular Maintenance, Damage Control, and Inflammatory Resolution in AN-Acute

### 8.1. Autophagy: Recycling, Survival, and Immune-Cell Maintenance

Autophagy is an intracellular recycling system that breaks down damaged proteins, mitochondria, and other cellular components so their constituents can be reused. During nutrient deprivation, this can provide metabolic substrates while removing damaged material that might otherwise impair cellular function or promote inflammation [87]. 

Autophagy also supports immune-cell survival and homeostasis. In T cells, it helps maintain mitochondrial quality, metabolic stability, and long-term survival and contributes to memory-cell formation. Autophagy similarly supports B-cell development and survival [88]. Thus, during prolonged energy restriction, autophagy could potentially help maintain existing T- and B-cell populations even when the greater metabolic investment required for activation and clonal expansion is constrained.

These functions may be particularly relevant in AN-acute because nutrient deprivation increases reliance on autophagic recycling. Additionally, exercise can stimulate autophagy and mitophagy, particularly in skeletal muscle, helping remove damaged proteins and mitochondria and support adaptation to repeated metabolic stress [89].

Under this hypothesis, severe caloric restriction and persistent HyAc may therefore increase reliance on autophagy to conserve limited substrates, maintain cellular integrity, and support the survival of existing immune cells.

### 8.2. Apoptosis: Controlled Removal of Damaged or Infected Cells

Apoptosis is a controlled form of programmed cell death that removes damaged, stressed, infected, or unnecessary cells while limiting inflammatory disruption [89]. Nutrient deprivation can shift cells from autophagic survival toward apoptosis when metabolic stress becomes more severe or prolonged, and acute strenuous exercise can also transiently increase apoptosis [90,91]. Short-term fasting may further amplify exercise-associated T-cell apoptosis [92].

In AN-acute, severe energy restriction combined with persistent activity could therefore increase cellular stress and favor apoptosis when damage exceeds the capacity for repair or autophagic recycling. However, direct evidence that apoptosis is increased in AN-acute is currently lacking, so this remains a proposed mechanism rather than an established finding.

### 8.3. Efferocytosis and Supporting Cellular Maintenance Mechanisms

Efferocytosis is the clearance of apoptotic cells by macrophages and other phagocytes. By removing dying cells before secondary necrosis occurs, it limits inflammatory spillover and promotes resolution and tissue repair [93].

In the present model, apoptosis and efferocytosis are considered secondary extensions of autophagy’s better-established role during nutrient deprivation. Together, these processes may support cellular maintenance and inflammatory resolution under metabolic stress. During severe caloric restriction, autophagy may promote cellular recycling and survival. When damage becomes irreversible, apoptosis removes cells in a controlled manner, followed by efferocytotic clearance that may limit secondary inflammation. Because nutrient deprivation and strenuous exercise can increase apoptosis in selected cell populations, these processes could help preserve tissue integrity while restraining inflammatory amplification. Direct evidence for altered apoptosis or efferocytosis in AN-acute is limited, and their contribution remains speculative.

## 9. Acute-Phase Response

The acute-phase response (APR) is a coordinated systemic reaction to infection or tissue injury, involving inflammatory cytokines that stimulate the production of acute-phase proteins, fever, and characteristic behavioral changes such as fatigue and reduced activity [71]. These responses support host defense but also require substantial metabolic resources. In AN-acute, several APR components appear attenuated. The present model proposes that the absence of an APR may contribute to an inflammation-restrained host-defense strategy under severe energy restriction and persistent HyAc.

### 9.1. Acute-Phase Proteins in AN-Acute

During systemic inflammation, cytokines stimulate the liver to increase acute-phase protein production that supports host defense, pathogen containment, tissue repair, and inflammation regulation. C-reactive protein (CRP) is one of the best-known positive acute-phase proteins. IL-6 primarily drives its hepatic synthesis, with additional modulation by cytokines such as IL-1β and TNF-α [71].

Importantly, CRP does not appear elevated in AN. A recent systematic review and meta-analysis found significantly lower circulating CRP concentrations in individuals with AN than in HCs [94]. This is notable because circulating IL-6 has frequently been reported as elevated in AN-acute. The combination of elevated IL-6 with low CRP suggests that IL-6 elevation alone should not be interpreted as evidence of a coordinated systemic APR. One possibility proposed in the present model is that some circulating IL-6 reflects skeletal muscle release associated with HyAc rather than with conventional inflammatory activation. However, the source and signaling characteristics of IL-6 in AN-acute remain unestablished.

A similar lack of inflammation is suggested by the erythrocyte sedimentation rate (ESR), an indirect marker of inflammation that rises partly because of acute-phase proteins such as fibrinogen. In a study of 327 adolescent girls with eating disorders and weight loss, ESR values were within normal reference ranges [95]. These findings do not suggest prominent systemic inflammatory activation.

Taken together, low CRP and normal ESR argue against interpreting isolated cytokine elevations as evidence of generalized systemic inflammation in AN-acute. Instead, these findings are consistent with the inflammation-restrained phenotype proposed in this review.

### 9.2. Thermoregulatory Adaptation in AN-Acute

#### 9.2.1. Fever as Part of the Acute-Phase Response

Fever is a major component of the acute-phase response (APR). During infection, pyrogenic cytokines promote prostaglandin E_2_ signaling in the hypothalamus, raising the thermoregulatory set point and core temperature. Fever-range temperatures can enhance leukocyte trafficking and selected innate and adaptive immune functions while also restricting the replication of some pathogens [96].

Fever is also metabolically expensive. A 1 °C rise in core temperature is associated with a 10–12% increase in metabolic rate. Generating and sustaining fever may therefore be particularly costly in AN-acute, where energy availability is already severely constrained. Consistent with this, attenuated febrile responses have been reported during bacterial infection in AN [13,97].

The combination of a reduced conventional febrile response and a relatively low burden of clinically apparent infection suggests that non-febrile warming may intermittently achieve some temperature-dependent immune effects. Experimental evidence indicates that some immune effects of fever-range heat depend partly on the temperature attained and duration of exposure, rather than solely on the signaling pathways that generate fever [96]. Physical activity as well as passive heating may provide one such source of warming.

#### 9.2.2. Activity-Induced Warming: Intermittent Fever-like Thermal Exposure

Skeletal-muscle metabolism is highly thermogenic. More than 75% of the energy generated during substrate oxidation is released as heat [98]. In healthy individuals, sustained exercise can raise core temperature by one or more degrees and, during strenuous activity, sometimes into the mild fever range [99].

This capacity is retained in AN-acute. Davies et al. showed that 60 min of submaximal cycling substantially increased rectal temperature in individuals with AN despite their low resting temperature; they were able to reach values that overlapped with mild fever [43]. Although exercise-induced warming is not true fever because the hypothalamic set point is unchanged, immune cells are nevertheless exposed to the attained temperature. Repeated bouts of ExEx and NEAT in AN-acute could therefore produce intermittent fever-like thermal exposure and reproduce selected temperature-dependent immune effects. This remains untested.

Passive thermoregulation may also be important. During the Minnesota Starvation Experiment, semi-starved subjects became markedly cold-sensitive and sought warmth through extra clothing, blankets, radiators, hot showers, sunlight, and hot food or beverage [100]. Similar behaviors have been reported in AN. Luck and Wakeling [101] provided direct evidence of altered heat-seeking behavior by showing that a subgroup of malnourished patients with AN preferred significantly warmer thermal stimuli than HCs. These observations suggest that severe energy deprivation can generate a strong drive to conserve or obtain external heat, although they do not establish that passive warming raises core temperature into the fever range.

An evolutionary parallel is seen in ectotherms. In infected desert iguanas, selecting warmer environments raised body temperature by about 2 °C and improved survival, whereas preventing this rise reduced survival [102]. Although this is not equivalent to passive warming in humans, it shows that behaviorally acquired heat can influence host defense.

Researchers have studied the thermoregulatory role of activity extensively in the ABA model. Food restriction combined with wheel access produces progressive weight loss, hypothermia, and hyperactivity. Gutiérrez and colleagues proposed that running may serve, at least in part, as an active thermoregulatory response, generating heat that helps counteract falling body temperature [103,104,105,106]. 

Complementary evidence from food-restricted mice suggests that exercise-induced warming remains effective during severe nutritional restriction. Filteau et al. [107] provide particularly relevant evidence that exercise-induced warming is retained during severe nutritional restriction. Mice were either fed ad libitum or subjected to food restriction that resulted in a loss of approximately 25% of their initial body weight over three weeks. Half of each group performed treadmill exercise for 30 minutes per day, five days per week. The published temperature figure shows that food restriction progressively lowered resting body temperature, whereas exercise produced marked elevations in body temperature in the food-restricted mice. Visual inspection of the graph indicates that post-exercise temperature approached 39–40 °C, comparable to values observed in the ad libitum-fed mice. Although this was not an ABA model, the study provides direct experimental evidence that exercise-induced warming remains effective during severe nutritional restriction.

Alongside activity-generated heat, ABA animals also use passive warmth when it is available. Increasing the ambient temperature reduces wheel running, limits weight loss, and promotes recovery [26,104]. In addition, access to a heated surface prevents hypothermia and reduces wheel running and weight loss [108], while housing at approximately 32 °C markedly reduces hypothermia, hyperactivity, and loss of body and muscle mass [63]. Together, these findings suggest that external warmth can reduce the thermoregulatory drive for activity.

It remains unclear how much core temperature rises during individual bouts of ABA running and whether these transient increases reach the mild fever range. Similarly, it is unclear whether passive warming produces comparable temperature excursions. Continuous core-temperature telemetry during running and passive warming would directly test whether either form of thermoregulation produces intermittent fever-like thermal exposure.

#### 9.2.3. Resting Low Core Temperature: Energy Conservation and Immune Restraint

At rest, individuals with AN-acute commonly exhibit reduced core temperature and marked cold intolerance [109,110], sometimes reaching clinically significant hypothermia [96]. This likely reflects starvation-related reductions in metabolic rate and heat production, together with loss of insulating adipose tissue.

Lower temperature can also restrain immune function. Mild experimental hypothermia reduces selected neutrophil antimicrobial functions, including reactive oxygen production and, in some studies, phagocytosis [111]. In this context, its significance may therefore lie less in enhancing pathogen clearance than in reducing metabolic demand and limiting energetically costly inflammatory activity.

This distinction aligns with experimental evidence suggesting that fever and hypothermia represent contrasting physiological responses to severe infection. Fever can enhance antimicrobial defense but is energetically costly, whereas hypothermia may preserve host function under severe metabolic stress [112]. In rats with severe LPS- or *Escherichia coli*-induced systemic inflammation, spontaneous hypothermia was associated with reduced organ dysfunction and improved survival despite a greater bacterial burden in the liver [113]. Thus, improved host survival did not necessarily require more effective pathogen clearance.

Within this hypothesis, resting hypothermia in AN-acute may therefore contribute to an energy-conserving, disease-tolerant phenotype characterized by reduced inflammatory expenditure. This possibility remains speculative and has not been directly demonstrated in AN.

#### 9.2.4. Proposed Thermal Cycling in AN-Acute

The present model tentatively proposes two complementary thermal states in AN-acute: a low resting core temperature that conserves energy but may constrain selected immune functions, and intermittent behaviorally induced warming that may transiently support temperature-dependent immune processes. This alternation, termed thermal cycling here, may arise from a combination of severe energy restriction, resting hypothermia, and persistent HyAc rather than a specific immune adaptation.

Initially, activity-induced warming, as an alternative to sustained fever, may seem contradictory, since physical activity, like fever, is energetically costly. However, HyAc in AN-acute serves multiple functions simultaneously, including food-seeking behavior, mood and anxiety modulation, thermogenesis, substrate mobilization, and myokine signaling. In this context, the energetic cost of movement may yield broader physiological benefits than heat production alone. 

Whether thermal cycling occurs and meaningfully influences immune function in AN-acute remains to be tested.

### 9.3. Sickness Behavior, Starvation Lethargy, and Recovery in AN-acute

Semi-starved individuals commonly become lethargic and less physically active; however, this should not necessarily be interpreted as classical sickness behavior. In uncomplicated starvation, reduced activity more likely reflects reduced metabolic energy availability and thus energy conservation, including reduced thyroid activity, diminished energy availability, loss of muscle mass, and other adaptations that lower energy expenditure [114].

In contrast, many individuals with AN-acute remain restless and physically active despite profound caloric restriction [115]. This relative absence of lethargy could suggest attenuated sickness behavior, particularly because fever and marked acute-phase responses also appear limited. 

However, HyAc may instead arise from starvation-related neural, metabolic, and thermoregulatory mechanisms, potentially influenced by genetic predisposition, that are distinct from classical sickness-behavior pathways [34,116]. Thus, persistent HyAc should not by itself be interpreted as evidence that sickness behavior is absent.

Another issue concerns how infection is subjectively experienced in AN-acute. If severe undernutrition attenuates the APR, an infected individual may experience fewer noticeable sickness-related symptoms, such as fatigue, lethargy, malaise, or fever, and may therefore be less likely to feel unwell.

However, nutritional rehabilitation can improve immune responsiveness, including monocyte production of IL-1, IL-6, and TNF-α, cytokines involved in generating sickness behavior [116,117]. Weight restoration could therefore increase the capacity to express more typical infection-related symptoms. If so, previously muted fatigue, malaise, lethargy, or fever might become more apparent and contribute to reports of feeling “less well” during recovery. This interpretation remains untested and requires direct investigation.

### 9.4. Disease Tolerance in AN-Acute

The combined findings of reduced febrile responses, low resting temperature, and restrained systemic inflammation raise a broader question: whether AN-acute may rely more heavily on disease tolerance as a host-defense strategy.

Disease tolerance differs from pathogen resistance. Whereas resistance mechanisms act primarily to reduce pathogen burden, tolerance mechanisms limit the physiological and tissue damage caused by both pathogens and the host’s inflammatory response, thereby preserving host function without necessarily reducing pathogen load [118,119].

In the present model, reduced febrile signaling, low resting temperature, restrained systemic inflammation, and constrained adaptive immune expansion may collectively reflect greater reliance on disease tolerance. This strategy could be particularly relevant under severe energy restriction, because inflammatory amplification, fever, and large adaptive responses carry substantial metabolic and tissue costs.

Disease tolerance may also help explain reports of bacterial infection in AN-acute accompanied by remarkably limited fever or other clinical signs [120] These observations do not establish disease tolerance in AN-acute, but they are consistent with the possibility that preserving physiological function while limiting inflammatory costs becomes increasingly important when energy availability is severely constrained.

The question, therefore, is not simply why systemic inflammatory responses appear muted, but how the body manages microbial challenges when systemic inflammatory responses, including pro-inflammatory cytokines, fever, and acute-phase proteins, are restrained. This leads directly to the innate defenses considered next, particularly neutrophils, macrophages, and dendritic cells.

## 10. Innate Immune Defenses in AN-Acute

Innate immunity provides a rapid, first-line defense against invading pathogens through cells such as neutrophils (PMNs), macrophages, dendritic cells (DCs), and natural killer (NK) cells, as well as soluble defenses such as cytokines and complement. These defenses recognize conserved microbial or damage signals and can often contain or eliminate pathogens before the adaptive immune response is needed.

In healthy controls, when early pathogen containment is insufficient, innate immune cells, particularly dendritic cells (DCs), help initiate the slower, antigen-specific adaptive immune response [50]. This hypothesis proposes that AN-acute may retain sufficient innate function for early containment, while limited energy availability constrains progression to a larger adaptive response when needed.

### 10.1. Neutrophils: Front-Line Antimicrobial Defense in AN-Acute

Neutrophils are the most abundant circulating leukocytes and are among the first immune cells recruited during acute infection. Although they can cause collateral tissue damage, they also contribute to wound healing and inflammatory resolution. Contemporary research has reframed neutrophils from simple, destructive cells to highly adaptable participants in host defense. They eliminate microorganisms through phagocytosis, release antimicrobial granules, produce reactive oxygen species (ROS), and form neutrophil extracellular traps (NETs). They also interact with macrophages, DCs, and other immune cells, contributing to pathogen clearance and to the regulation and resolution of inflammation [121].

Severe starvation can impair neutrophil biology without necessarily eliminating front-line innate defense. In AN, leukopenia and neutropenia are well recognized, particularly with greater nutritional severity, and bone-marrow hypoplasia or gelatinous transformation may further reduce neutrophil production and reserve [122]. Functional impairments have also been reported. Gotch et al. [123] demonstrated reduced granulocyte bactericidal activity in severely malnourished individuals with AN, with improvement following nutritional rehabilitation. Other studies describe abnormalities in selected neutrophil functions, although findings have been inconsistent [7]. Thus, starvation may reduce neutrophil numbers, reserves, and selected antimicrobial functions without necessarily producing a complete failure of front-line defense.

Neutrophils also have a metabolic profile that may be particularly relevant to AN-acute. They rely heavily on glycolysis for rapid energy production and can mobilize intracellular glycogen stores when extracellular fuel is limited. Experimental work indicates that glycogen synthesis and mobilization support neutrophil survival and bacterial killing under metabolically restricted conditions [124]. This metabolic flexibility could help sustain selected antimicrobial functions during nutrient limitation, although its importance has not been directly demonstrated in AN-acute.

Physical activity may further influence neutrophil availability. Acute exercise rapidly increases circulating neutrophil numbers by mobilizing them from vascular and bone-marrow pools, with stress hormones contributing to this response. Exercise can also transiently alter neutrophil functional activity, although these responses depend on exercise intensity, duration, and recovery [125].

In AN-acute, repeated bouts of activity or prolonged low-intensity movement could therefore repeatedly mobilize existing neutrophils into circulation. The hypothesis is not that HyAc makes neutrophils unusually effective, but that it may provide a partial “salvage” effect: starvation reduces neutrophil numbers and reserves, but repeated movement may temporarily increase the circulating availability of the remaining cells. Their predominantly glycolytic metabolism and ability to generate and mobilize intracellular glycogen may further help sustain rapid antimicrobial responses when extracellular fuel availability is limited [124].

Thus, PMNs may remain a critical component of rapid innate defense during severe energy restriction. Their numbers and some functions may be impaired, but metabolic flexibility together with repeated activity-related mobilization could help preserve sufficient front-line antimicrobial capacity for early pathogen containment. Whether this occurs in AN-acute remains hypothetical and requires direct study of neutrophil numbers, mobilization, antimicrobial function, and metabolism, ideally before and after controlled physical activity.

### 10.2. Macrophages: Pathogen Defense, Inflammatory Resolution, and Exercise

Macrophages are highly adaptable innate immune cells whose functions change in response to pathogens, metabolic conditions, and local cytokine signals. During acute infection, they can adopt more inflammatory functions and produce cytokines such as TNF-α, IL-1β, and IL-6, contributing to pathogen defense, the acute-phase response, fever, and sickness behavior [116]. As the threat is controlled, macrophage activity can shift toward resolution, including clearance of apoptotic cells through efferocytosis, restraint of excessive inflammation, and tissue repair [126,127].

Exercise can also influence macrophage function. Acute strenuous or tissue-damaging exercise may transiently increase inflammatory signaling, whereas repeated moderate activity is associated with a more inflammation-limiting systemic environment [36]. In experimental metabolic-tissue models, exercise training can reduce macrophage infiltration and shift the phenotype away from strongly inflammatory states toward more repair-oriented states [128]. These findings do not prove a universal M1-to-M2 transition across tissues, but they show that macrophage function responds to repeated physical activity.

This flexibility may be particularly relevant to AN-acute. Persistent physical activity occurs within a metabolic environment characterized by severe energy restriction, elevated cortisol and adiponectin, increased ketone availability, and repeated muscle-derived signaling. Glucocorticoids strongly restrain inflammatory gene expression and immune activation [76] while β-hydroxybutyrate can suppress NLRP3 inflammasome activation [84]. Adiponectin has also been associated with inflammation-limiting effects on macrophage signaling. In this hypothesis, these signals may collectively bias macrophage activity away from sustained inflammatory amplification and toward more pro-resolving functions.

Importantly, pro-resolving activity does not imply loss of antimicrobial capacity. Macrophage phenotypes exist along a continuum rather than as fixed M1 or M2 states [129]. Macrophages can therefore retain phagocytic and clearance functions while simultaneously limiting unnecessary inflammatory amplification and promoting resolution.

Thus, PMNs and macrophages may provide sufficient early innate containment. However, escalation to a substantial adaptive response requires dendritic cell activation and maturation to initiate antigen-specific T-cell responses, along with B-cell activation and antibody production. The activation and expansion of these lymphocytes may represent a critical point of metabolic constraint in AN-acute.

### 10.3. Dendritic Cells: The Critical Bridge Between Innate and Adaptive Immunity

Dendritic cells (DCs) are specialized innate immune cells that continuously survey tissues for microbes, foreign antigens, and signs of cellular damage. In their relatively immature or steady-state, they focus on antigen sampling and local surveillance without necessarily initiating a strong adaptive response. With appropriate activation signals, DCs mature, migrate to lymphoid tissues, and become highly effective antigen-presenting cells that can initiate antigen-specific T-cell responses [130,131].

DCs are not typically reported as a distinct category in routine white blood cell counts. They are relatively uncommon in peripheral blood, whereas substantial populations reside in tissues and lymphoid organs [132]. Routine leukocyte counts in AN-acute therefore provide little information about DC abundance, activation state, or functional capacity.

#### 10.3.1. From Surveillance to Activation: A Metabolic Transition

DC activation involves substantial metabolic reprogramming. After recognizing sufficiently strong microbial or danger signals, DCs increase glycolysis and anabolic activity to support maturation, cytokine production, co-stimulatory molecule expression, migration, and effective T-cell stimulation [133].

This distinction may be particularly relevant to AN-acute. Neutrophils are highly glycolytic and can mobilize intracellular glycogen to support survival and antimicrobial function when extracellular substrates are limited [124]. By contrast, DCs must make an additional metabolic investment when moving from relatively economical surveillance to an activated, immunogenic state.

Severe energy restriction could therefore make this transition more difficult when substrates and anabolic resources are limited. Under this hypothesis, the problem may not be DCs’ failure to detect pathogens. Rather, the metabolic transition from surveillance to full immunogenic activation may be constrained. Severe energy restriction could raise the threshold for DC maturation and effective T-cell stimulation while permitting continued antigen sampling and innate surveillance. This mechanism has not been directly demonstrated in AN-acute.

#### 10.3.2. Activation of CD4+ Helper T Cells: The Bridge to Adaptive Immunity

Following activation, mature DCs migrate to lymph nodes and provide the signals needed to activate naïve T cells. Productive T-cell activation requires antigen recognition through the T-cell receptor, along with co-stimulatory signaling, while cytokines from DCs and the surrounding inflammatory environment further determine the magnitude and differentiation of the response. These are commonly described as signals 1 (antigen recognition), 2 (co-stimulation), and 3 (cytokine instruction) [134,135].

Once activated, CD4+ T cells proliferate and coordinate multiple components of adaptive immunity, including B-cell antibody responses and effective CD8+ T-cell responses. This expansion is metabolically demanding. Naïve T cells are comparatively metabolically quiescent, whereas activation produces a marked increase in nutrient uptake, glycolysis, biosynthesis, and cellular growth to support differentiation and rapid clonal expansion [52].

The DC–CD4+ axis may therefore contain two linked metabolic checkpoints in AN-acute. First, DCs must have sufficient metabolic resources to mature and provide effective antigen presentation, co-stimulation, and cytokine signaling. Second, activating CD4+ T cells must have sufficient resources for the metabolic reprogramming, growth, and proliferation required for clonal expansion [51].

**Figure 3:**
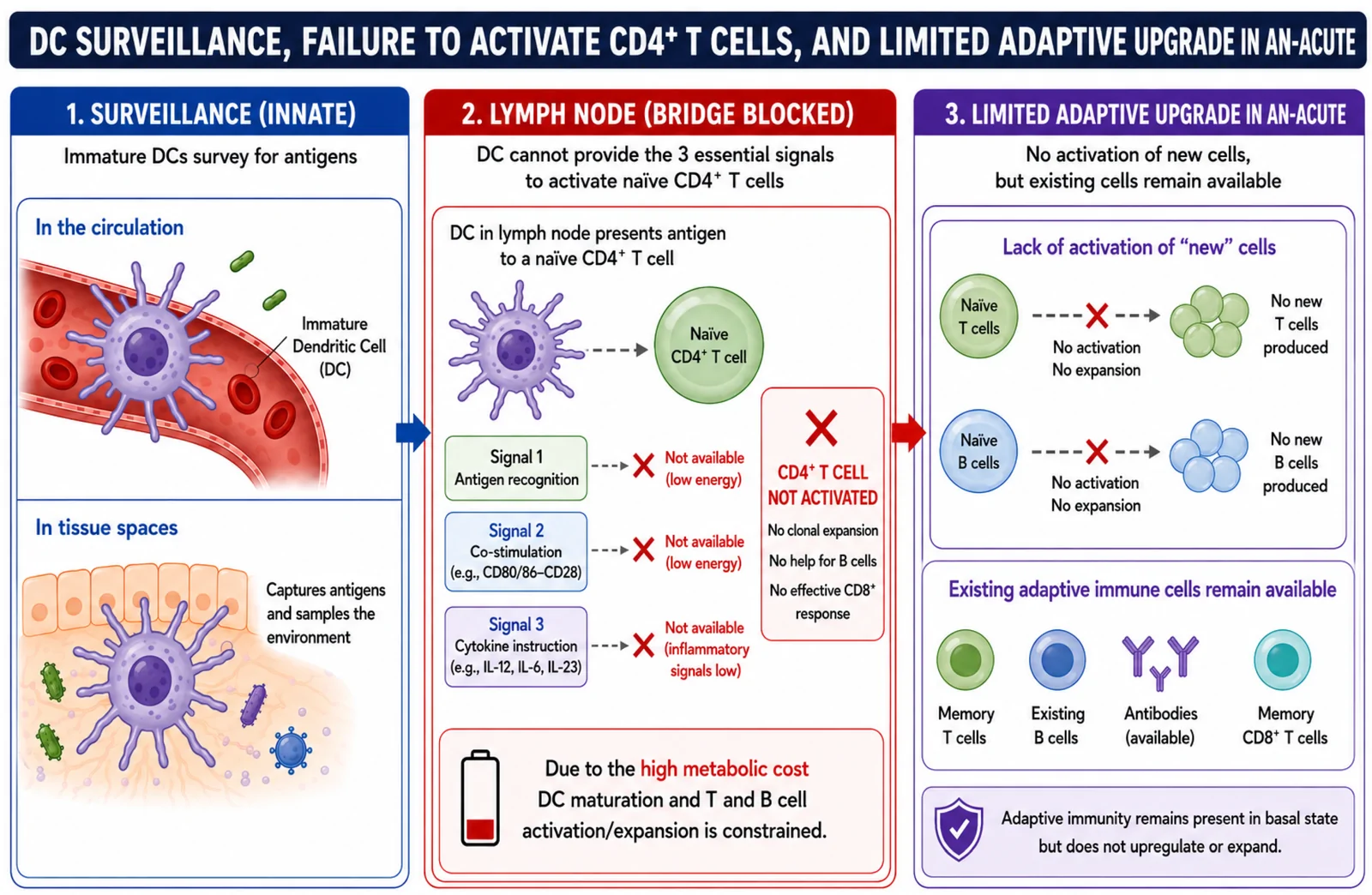
Proposed DC–CD4+ metabolic bottleneck in AN-acute, allowing continued dendritic-cell surveillance while constraining CD4+ T-cell activation and adaptive immune expansion.

Severe energy restriction could constrain either checkpoint without eliminating the lymphocytes already present (Figure 3). Naïve and memory T cells can persist in comparatively quiescent metabolic states, whereas activation and rapid expansion require greater nutrient and biosynthetic support [52]. The presence of circulating adaptive immune cells in AN-acute therefore does not establish that their capacity for full activation and expansion is preserved.

#### 10.3.3. What Is Known About DCs in AN-Acute?

Direct evidence regarding DC function in AN is limited. Pászthy et al. [66] found no generalized reduction in circulating myeloid or plasmacytoid DC populations. However, preserved cell numbers do not establish that DC maturation or T-cell activation capacity is intact.

Related work from the same group reported altered lymphocyte activation responses, including delayed intracellular calcium signaling and a lower proportion of IL-2-positive CD4+ cells following stimulation. These findings illustrate that preserved cell numbers do not necessarily imply preserved activation capacity. Broader reviews similarly describe heterogeneous findings in lymphocyte populations and function and emphasize the limited information available regarding DC function in AN [7].

Importantly, existing studies have not directly tested the complete sequence proposed here: DC metabolic activation → maturation and migration → effective co-stimulation → CD4+ T-cell activation → clonal expansion.

The present hypothesis is therefore not that DCs or lymphocytes are absent or globally defective in AN-acute. Rather, severe metabolic constraint may permit continued DC surveillance while limiting the transition to full immunogenic activation and subsequent CD4+ expansion. This proposed DC–CD4+ metabolic bottleneck could restrict adaptive upregulation while leaving substantial innate defense available. Whether such a bottleneck occurs in AN-acute remains unknown and represents an important testable prediction of the present model.

## 11. Summary of the Proposed Immune Hypothesis (Figure 4)

The present model integrates established physiological and immunologic findings in AN-acute with mechanisms that remain untested. It proposes that the immune phenotype reflects not generalized immune failure but a selective reorganization of host defense under prolonged energy constraint and persistent hyperactivity. Severe caloric restriction, compounded by HyAc, may reduce leptin signaling, increase AMPK activity, and suppress mTORC1-dependent anabolic processes. These changes may favor cellular survival and energy conservation while making metabolically expensive adaptive immune activation more difficult.

In this environment, muscle-derived IL-6 signaling associated with repeated activity, along with elevated cortisol, adiponectin, and ketone bodies, may promote fuel mobilization while restraining excessive inflammatory amplification. These signals may also support cellular maintenance and resolution processes, including autophagy and, potentially, apoptosis followed by efferocytosis. The resulting immune environment may therefore favor preservation, repair, and controlled inflammation rather than maximal systemic activation.

This restraint may extend to aspects of the acute-phase response, including lower acute-phase protein responses and attenuated fever. Persistent HyAc may arise through distinct neural, metabolic, and thermoregulatory mechanisms. However, as weight and immunity recover, the capacity to express more typical sickness-related symptoms during infection may increase. This could contribute to reports of feeling “less well” during weight restoration, although this possibility remains untested.

At the same time, selected innate defenses may remain relatively resilient. Neutrophils are highly glycolytic and can mobilize intracellular glycogen, while macrophages can combine antimicrobial clearance with pro-resolving functions. Persistent HyAc may further mobilize innate cells and provide repeated autonomic, myokine, and thermal signals. Thus, PMNs and macrophages may retain sufficient capacity to contain pathogens early, despite restrained systemic inflammation.

The principal metabolic bottleneck may occur at the transition from innate to adaptive immunity. DCs may continue antigen surveillance, while full maturation, migration, co-stimulation, and subsequent CD4+ T-cell activation require substantial metabolic reprogramming. A second constraint may arise during T-cell clonal expansion, which requires increased nutrient uptake, glycolysis, biosynthesis, and mTORC1-dependent growth. The proposed result is a DC–CD4+ metabolic bottleneck that limits adaptive escalation without eliminating the adaptive immune cells already present.

Taken together, the model predicts energy conservation, selective preservation of innate containment, restrained inflammatory amplification, and constrained adaptive upregulation. Some microbial challenges may be contained early. Others may remain relatively “silent” because pro-inflammatory cytokines, fever, and other acute-phase responses are attenuated. In some circumstances, host protection may rely more heavily on disease tolerance than on complete pathogen elimination.

This hypothesis does not propose that AN-acute confers superior immunity. Rather, under extreme metabolic constraint, the immune system may prioritize functions immediately necessary for survival while limiting prolonged, energetically costly, or tissue-damaging responses. Persistent HyAc may help shape this state. The model is testable by measuring neutrophil and macrophage function, DC–CD4+ activation, acute-phase responses, and changes during nutritional restoration and controlled physical activity.

## 12. Testable Predictions

The present hypothesis can be tested by comparing immune and physiological responses in AN-acute with those of healthy controls and by following patients during nutritional recovery.

First, studies should determine whether low-intensity movement elicits a meaningful physiological response in AN-acute. Continuous monitoring of activity, heart rate, HRV, and temperature, together with blood measurements before and after a standardized walking task, could show whether mild movement produces repeated exercise-like effects.

Second, the model predicts that innate immune defenses are relatively preserved, particularly neutrophil and macrophage functions involved in early pathogen containment and inflammatory resolution.

Third, researchers should directly test the proposed dendritic-cell–CD4+ bottleneck. The key question is whether dendritic cells can maintain basic immune surveillance while having greater difficulty fully activating and stimulating CD4+ T cells during severe energy restriction.

Finally, studies should examine whether adaptive immune responses and the acute-phase response strengthen with nutritional restoration, using measures such as vaccine responses, delayed-type hypersensitivity, fever, and pro-inflammatory markers.

Together, these studies could determine whether AN-acute is characterized by generalized immune impairment or, as proposed here, by preserved innate containment, restrained systemic inflammation, and constrained adaptive upregulation.

## 13. Conclusions

The apparent immune paradox in AN-acute is that, despite profound undernutrition and reported immune abnormalities, individuals have historically been described as experiencing fewer clinically apparent infections than expected [12].

The present model treats this not as a true paradox but as an unresolved clinical observation. It remains unclear whether infections are genuinely less frequent or whether microbial challenges are contained or clinically expressed differently under severe energy restriction and persistent HyAc.

In summary, the proposed immune phenotype for AN-acute reflects a shift toward energy conservation, preserved innate containment, restrained inflammatory amplification, and constrained adaptive upregulation. Some infections may be contained early. Others may present with muted clinical signs. In some circumstances, host protection may rely more on disease tolerance.

The central question, therefore, may not be whether individuals with AN-acute experience fewer infections, but rather how they manage microbial challenges and how these challenges are clinically expressed when extreme energy restriction and sustained physical activity shape immune defenses.

## Figures and Tables

**Figure 4:**
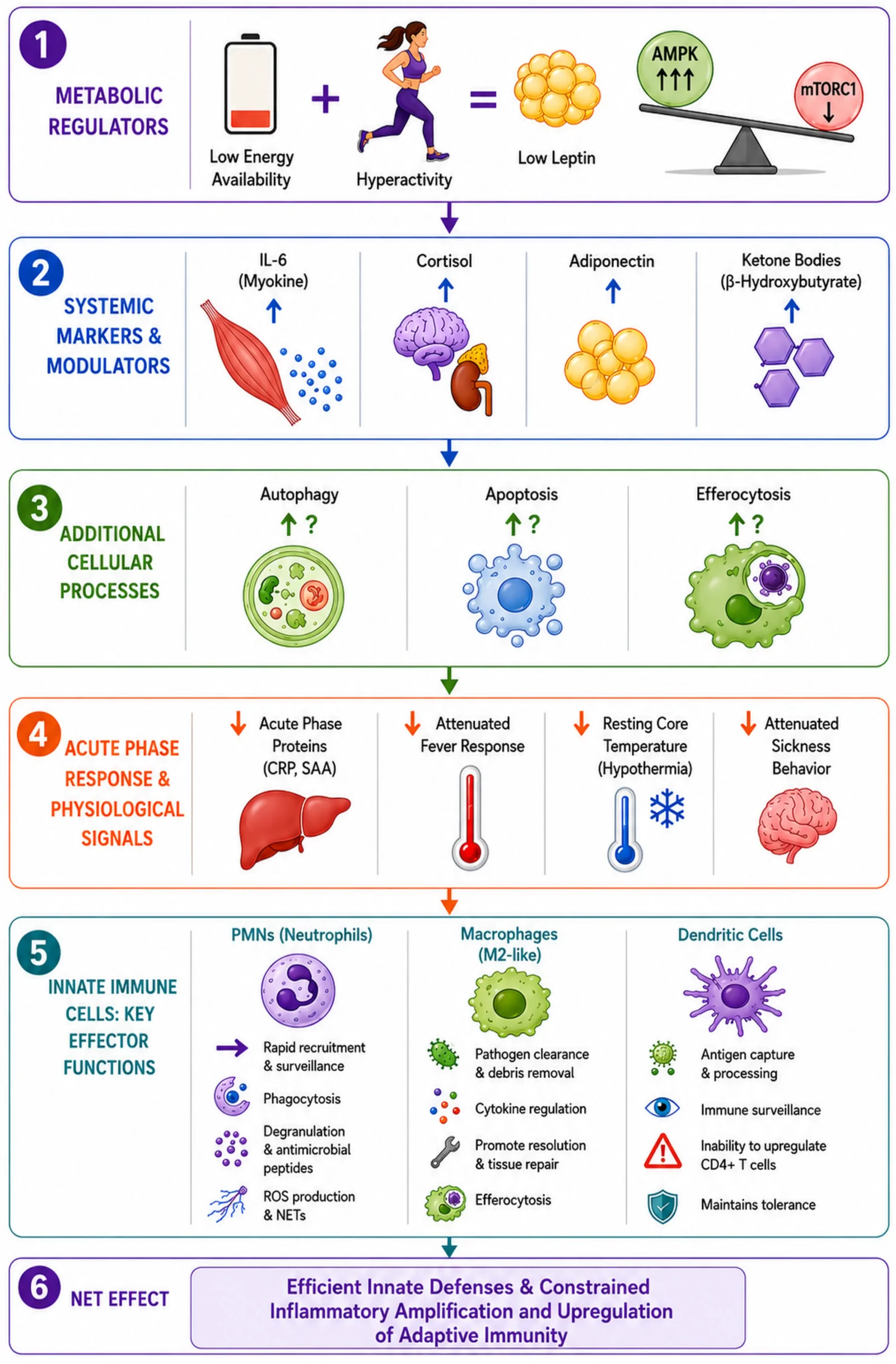
Summary of the proposed hypothesis illustrating how low caloric intake combined with hyperactivity (HyAc) may influence immune defense in AN-acute.

**Table 1:** Comparison of inflammation-associated IL-6 and contraction-induced muscle IL-6 myokine. Inflammation-associated IL-6 and muscle-derived IL-6 are the same protein. Their differing effects reflect their sources, stimuli, concentrations, durations, receptor pathways, and surrounding cytokine environments.

Feature	Inflammation-Associated IL-6	Contraction-Induced Muscle IL-6 Myokine	Selected References
Meaning of the term	IL-6 acts as an immune-signaling cytokine during infection, injury, or inflammation.	The same IL-6 molecule is released by contracting skeletal muscle; the term “myokine” denotes its muscular source.	[69]
Principal source	Mainly immune cells, including macrophages, monocytes, and fibroblasts.	Primarily contracting skeletal muscle fibers.	[69]
Principal stimulus	Pathogens, tissue damage, TNF-α, and IL-1β.	Muscle contraction, low muscle glycogen, increased energy demand, adrenergic stimulation.	[69]
Predominant signaling pattern	Persistent trans-signaling strongly associated with leukocyte recruitment and chronic inflammation.	More often associated with short-lived local and classic signaling.	[70]
Typical temporal pattern	Rises during infection or injury and may remain elevated as long as the inflammatory stimulus persists.	Rises during exercise, peaks near the end of or shortly after activity, then declines.	[69]
Duration of exposure	May be prolonged or chronic.	Usually brief, pulsatile, and transient.	[69]
Magnitude	Ranges from modest, persistent elevation to marked increases during severe infection or trauma.	May increase dramatically, sometimes approaching 100-fold during prolonged strenuous exercise.	[69]
Impact on metabolism	Persistent elevation may accompany insulin resistance and metabolic dysfunction.	Mobilizes fuel by increasing glucose availability, lipolysis, and fatty-acid oxidation.	[69]
Impact on inflammation	Can promote or maintain inflammation when persistent and combined with TNF-α, IL-1β, and chemokines.	Often has indirect anti-inflammatory effects by increasing IL-1ra, IL-10, and cortisol and reducing TNF-α.	[69]
Impact on innate and adaptive immunity	Supports innate defense and shapes adaptive responses; persistent signaling may contribute to autoimmune inflammation.	May help regulate innate inflammation and indirectly restrain excessive adaptive immune activation; direct effects remain context dependent.	[70]
Impact on acute-phase proteins	Sustained IL-6 strongly stimulates hepatic acute-phase protein production, including CRP.	Brief exercise-induced IL-6 may produce only limited or transient acute-phase responses.	[71]
Impact on fever	Contributes to fever via hypothalamic prostaglandin E_2_ signaling during inflammation.	Usually does not produce fever when released transiently during exercise.	[69]
Impact on sickness behavior	Together with IL-1β, TNF-α, and prostaglandins, it may contribute to fatigue, reduced appetite, lethargy, and social withdrawal.	Does not normally produce the sickness-behavior response associated with infection.	[69]

Abbreviations: IL-6, interleukin-6; CRP, C-reactive protein; IL-1β, interleukin-1 beta; IL-1ra, interleukin-1 receptor antagonist; IL-10, interleukin-10; TNF-α, tumor necrosis factor alpha.

## Data Availability

No new data were created or analyzed in this study. Data sharing is not applicable to this article.

## References

[1] Zipfel S., Giel K.E., Bulik C.M., Hay P., Schmidt U. (2015). Anorexia nervosa: Aetiology, assessment, and treatment. Lancet Psychiatry.

[2] Treasure J., Willmott D., Ambwani S., Cardi V., Bryan D.C., Rowlands K., Schmidt U. (2020). Cognitive Interpersonal Model for Anorexia Nervosa Revisited: The Perpetuating Factors that Contribute to the Development of the Severe and Enduring Illness. J. Clin. Med..

[3] Misra M., Miller K.K., Almazan C., Ramaswamy K., Aggarwal A., Herzog D.B., Neubauer G., Breu J., Klibanski A. (2004). Hormonal and Body Composition Predictors of Soluble Leptin Receptor, Leptin, and Free Leptin Index in Adolescent Girls with Anorexia Nervosa and Controls and Relation to Insulin Sensitivity. J. Clin. Endocrinol. Metab..

[4] Müller M.J., Enderle J., Bosy-Westphal A. (2016). Changes in Energy Expenditure with Weight Gain and Weight Loss in Humans. Curr. Obes. Rep..

[5] Keys A., Brozek J., Henschel A., Mickelsen O., Taylor H.L. (1950). The Biology of Human Starvation.

[6] Nova E., Marcos A. (2006). Immunocompetence to assess nutritional status in eating disorders. Expert Rev. Clin. Immunol..

[7] Gibson D., Mehler P.S. (2019). Anorexia Nervosa and the Immune System—A Narrative Review. J. Clin. Med..

[8] Solmi M., Veronese N., Favaro A., Santonastaso P., Manzato E., Sergi G., Correll C.U. (2015). Inflammatory cytokines and anorexia nervosa: A meta-analysis of cross-sectional and longitudinal studies. Psychoneuroendocrinology.

[9] Chandra R.K. (1997). Nutrition and the immune system: An introduction. Am. J. Clin. Nutr..

[10] Calder P.C., Jackson A.A. (2000). Undernutrition, infection and immune function. Nutr. Res. Rev..

[11] Armstrong-Esther C.A., Lacey J.H., Crisp A.H., Bryant T.N. (1978). An investigation of the immune response of patients suffering from anorexia nervosa. Postgrad. Med. J..

[12] Bowers T.K., Eckert E. (1978). Leukopenia in anorexia nervosa. Lack of increased risk of infection. Arch. Intern. Med..

[13] Brown R.F., Bartrop R., Birmingham C.L. (2008). Immunological disturbance and infectious disease in anorexia nervosa: A review. Acta Neuropsychiatr..

[14] Campbell J.P., Turner J.E. (2018). Debunking the Myth of Exercise-Induced Immune Suppression: Redefining the Impact of Exercise on Immunological Health Across the Lifespan. Front. Immunol..

[15] Nieman D.C., Wentz L.M. (2019). The compelling link between physical activity and the body’s defense system. J. Sport Health Sci..

[16] Machado R.B., Rocha M.R., Suchecki D. (2017). Brain prolactin is involved in stress-induced REM sleep rebound. Horm. Behav..

[17] Fernstrom M.H., Weltzin T.E., Neuberger S., Srinivasagam N., Kaye W.H. (1994). Twenty-four-hour food intake in patients with anorexia nervosa and in healthy control subjects. Biol. Psychiatry.

[18] Hadigan C.M., Anderson E.J., Miller K.K., Hubbard J.L., Herzog D.B., Klibanski A., Grinspoon S.K. (2000). Assessment of macronutrient and micronutrient intake in women with anorexia nervosa. Int. J. Eat. Disord..

[19] Jenkins P.E., Proctor K., Snuggs S. (2024). Dietary intake of adults with eating disorders: A systematic review and meta-analysis. J. Psychiatr. Res..

[20] Casper R.C., Voderholzer U., Naab S., Schlegl S. (2020). Increased urge for movement, physical and mental restlessness, fundamental symptoms of restricting anorexia nervosa?. Brain Behav..

[21] Gull W.W. (1997). Anorexia Nervosa (Apepsia Hysterica, Anorexia Hysterica). Obes. Res..

[22] Crisp A. (1967). The possible significance of some behavioural correlates of weight and carbohydrate intake. J. Psychosom. Res..

[23] Meyer C., Taranis L., Goodwin H., Haycraft E. (2011). Compulsive exercise and eating disorders. Eur. Eat. Disord. Rev..

[24] Touyz S., Hay P., Noetel M. (2017). Is the neglect of exercise in anorexia nervosa research a case of “running out” of ideas or do we need to take a “LEAP” of faith into the future?. J. Eat. Disord..

[25] Guisinger S. (2003). Adapted to flee famine: Adding an evolutionary perspective on anorexia nervosa. Psychol. Rev..

[26] Gutiérrez E., Vázquez R., Boakes R.A. (2002). Activity-based anorexia: Ambient temperature has been a neglected factor. Psychon. Bull. Rev..

[27] Scolnick B., Zupec-Kania B., Calabrese L., Aoki C., Hildebrandt T. (2020). Remission from Chronic Anorexia Nervosa with Ketogenic Diet and Ketamine: Case Report. Front. Psychiatry.

[28] Smith L.L. (2021). The Central Role of Hypothermia and Hyperactivity in Anorexia Nervosa: A Hypothesis. Front. Behav. Neurosci..

[29] O’Neill L.A., Kishton R.J., Rathmell J. (2016). A guide to immunometabolism for immunologists. Nat. Rev. Immunol..

[30] Buck M.D., Sowell R.T., Kaech S.M., Pearce E.L. (2017). Metabolic Instruction of Immunity. Cell.

[31] McDade T.W. (2003). Life history theory and the immune system: Steps toward a human ecological immunology. Am. J. Phys. Anthropol..

[32] Rauw W.M. (2012). Immune response from a resource allocation perspective. Front. Genet..

[33] Casper R.C. (2006). The ‘drive for activity’ and “restlessness” in anorexia nervosa: Potential pathways. J. Affect. Disord..

[34] Casper R.C. (2016). Restless activation and drive for activity in anorexia nervosa may reflect a disorder of energy homeostasis. Int. J. Eat. Disord..

[35] Spurway N., MacLaren D., Nieman D.C., Gleeson M. (2006). Immune Function in Sport and Exercise.

[36] Gleeson M., Bishop N.C., Stensel D.J., Lindley M.R., Mastana S.S., Nimmo M.A. (2011). The anti-inflammatory effects of exercise: Mechanisms and implications for the prevention and treatment of disease. Nat. Rev. Immunol..

[37] Levine J.A., Kotz C.M. (2005). NEAT—Non-exercise activity thermogenesis—Egocentric & geocentric environmental factors vs. biological regulation. Acta Physiol. Scand..

[38] Belak L., Gianini L., Klein D.A., Sazonov E., Keegan K., Neustadt E., Walsh B.T., Attia E. (2017). Measurement of fidgeting in patients with anorexia nervosa using a novel shoe-based monitor. Eat. Behav..

[39] Petretta M., Bonaduce D., Themistoclakis S., Ianniciello A., Scalfi L., De Filippo E., Contaldo F., Marciano F., Migaux M. (1997). Heart rate variability as a measure of autonomic nervous system function in anorexia nervosa. Clin. Cardiol..

[40] Peyser D., Scolnick B., Hildebrandt T., Taylor J.A. (2020). Heart rate variability as a biomarker for anorexia nervosa: A review. Eur. Eat. Disord. Rev..

[41] Billeci L., Tonacci A., Brunori E., Raso R., Calderoni S., Maestro S., Morales M.A. (2019). Autonomic Nervous System Response during Light Physical Activity in Adolescents with Anorexia Nervosa Measured by Wearable Devices. Sensors.

[42] Kolnes L.-J. (2016). ‘Feelings stronger than reason’: Conflicting experiences of exercise in women with anorexia nervosa. J. Eat. Disord..

[43] Davies C.T.M., Fohlin L., Thorén C. (1979). Temperature Regulation in Anorexia Nervosa Patients during Prolonged Exercise. Acta Medica Scand..

[44] Schalla M.A., Stengel A. (2019). Activity Based Anorexia as an Animal Model for Anorexia Nervosa–A Systematic Review. Front. Nutr..

[45] Medzhitov R. (2007). Recognition of microorganisms and activation of the immune response. Nature.

[46] Flajnik M.F., Kasahara M. (2009). Origin and evolution of the adaptive immune system: Genetic events and selective pressures. Nat. Rev. Genet..

[47] Medzhitov R. (2010). Innate immunity: Quo vadis?. Nat. Immunol..

[48] Bonilla F.A., Oettgen H.C. (2010). Adaptive immunity. J. Allergy Clin. Immunol..

[49] Heinzel S., Marchingo J.M., Horton M.B., Hodgkin P.D. (2018). The regulation of lymphocyte activation and proliferation. Curr. Opin. Immunol..

[50] Liu J., Zhang X., Cheng Y., Cao X. (2021). Dendritic cell migration in inflammation and immunity. Cell. Mol. Immunol..

[51] Nieman D.C., Pence B.D. (2020). Exercise immunology: Future directions. J. Sport Health Sci..

[52] Chapman N.M., Boothby M.R., Chi H. (2020). Metabolic coordination of T cell quiescence and activation. Nat. Rev. Immunol..

[53] Hebebrand J., Blum W.F., Barth N., Coners H., Englaro P., Juul A., Ziegler A., Warnke A., Rascher W., Remschmidt H. (1997). Leptin levels in patients with anorexia nervosa are reduced in the acute stage and elevated upon short-term weight restoration. Mol. Psychiatry.

[54] Misra M., Klibanski A. (2014). Endocrine consequences of anorexia nervosa. Lancet Diabetes Endocrinol..

[55] Misra M. (2014). Neuroendocrine mechanisms in athletes. Handbook of Clinical Neurology.

[56] Lord G.M., Matarese G., Howard J.K., Baker R.J., Bloom S.R., Lechler R.I. (1998). Leptin modulates the T-cell immune response and reverses starvation-induced immunosuppression. Nature.

[57] Saucillo D.C., Gerriets V.A., Sheng J., Rathmell J.C., MacIver N.J. (2014). Leptin Metabolically Licenses T Cells for Activation To Link Nutrition and Immunity. J. Immunol..

[58] Naylor C., Petri W.A.J. (2016). Leptin Regulation of Immune Responses. Trends Mol. Med..

[59] Hardie D.G., Ross F.A., Hawley S.A. (2012). AMPK: A nutrient and energy sensor that maintains energy homeostasis. Nat. Rev. Mol. Cell Biol..

[60] O’Neill L.A.J., Hardie D.G. (2013). Metabolism of inflammation limited by AMPK and pseudo-starvation. Nature.

[61] Saxton R.A., Sabatini D.M. (2017). mTOR Signaling in Growth, Metabolism, and Disease. Cell.

[62] Wei J., Raynor J., Nguyen T.-L.M., Chi H. (2017). Nutrient and Metabolic Sensing in T Cell Responses. Front. Immunol..

[63] Fraga A., Rial-Pensado E., Nogueiras R., Fernø J., Diéguez C., Gutierrez E., López M. (2021). Activity-Based Anorexia Induces Browning of Adipose Tissue Independent of Hypothalamic AMPK. Front. Endocrinol..

[64] Procaccini C., De Rosa V., Galgani M., Carbone F., Cassano S., Greco D., Qian K., Auvinen P., Calì G., Stallone G. (2012). Leptin-Induced mTOR Activation Defines a Specific Molecular and Transcriptional Signature Controlling CD4+ Effector T Cell Responses. J. Immunol..

[65] Gwinn D.M., Shackelford D.B., Egan D.F., Mihaylova M.M., Mery A., Vasquez D.S., Turk B.E., Shaw R.J. (2008). AMPK Phosphorylation of Raptor Mediates a Metabolic Checkpoint. Mol. Cell.

[66] Pászthy B., Švec P., Vásárhelyi B., Túry F., Mazzag J., Tulassay T., Treszl A. (2007). Investigation of regulatory T cells in anorexia nervosa. Eur. J. Clin. Nutr..

[67] Nagata T., Kiriike N., Tobitani W., Kawarada Y., Matsunaga H., Yamagami S. (1999). Lymphocyte subset, lymphocyte proliferative response, and soluble interleukin-2 receptor in anorexic patients. Biol. Psychiatry.

[68] Keeler J.L., Bovenberg C., Himmerich H., Treasure J., Carter B., Schmidt U., Dalton B. (2025). Cytokine concentrations in people with eating disorders: A comprehensive updated systematic review and meta-analysis. Commun. Med..

[69] Pedersen B.K., Febbraio M.A. (2008). Muscle as an Endocrine Organ: Focus on Muscle-Derived Interleukin-6. Physiol. Rev..

[70] Rose-John S., Jenkins B.J., Garbers C., Moll J.M., Scheller J. (2023). Targeting IL-6 trans-signalling: Past, present and future prospects. Nat. Rev. Immunol..

[71] Gabay C., Kushner I. (1999). Acute-Phase Proteins and Other Systemic Responses to Inflammation. New Engl. J. Med..

[72] Carey A.L., Steinberg G.R., Macaulay S.L., Thomas W.G., Holmes A.G., Ramm G., Prelovsek O., Hohnen-Behrens C., Watt M.J., James D.E. (2006). Interleukin-6 Increases Insulin-Stimulated Glucose Disposal in Humans and Glucose Uptake and Fatty Acid Oxidation In Vitro via AMP-Activated Protein Kinase. Diabetes.

[73] Thavaraputta S., Ungprasert P., Witchel S.F., Fazeli P.K. (2023). Anorexia nervosa and adrenal hormones: A systematic review and meta-analysis. Eur. J. Endocrinol..

[74] Hackney A.C. (2006). Stress and the neuroendocrine system: The role of exercise as a stressor and modifier of stress. Expert Rev. Endocrinol. Metab..

[75] Burden V.R., White B.D., Dean R.G., Martin R.J. (1993). Activity of the Hypothalamic-Pituitary-Adrenal Axis is Elevated in Rats with Activity-Based Anorexia. J. Nutr..

[76] Cain D.W., Cidlowski J.A. (2017). Immune regulation by glucocorticoids. Nat. Rev. Immunol..

[77] Kuo T., Harris C.A., Wang J.-C. (2013). Metabolic functions of glucocorticoid receptor in skeletal muscle. Mol. Cell. Endocrinol..

[78] Kuo T., McQueen A., Chen T.-C., Wang J.-C. (2015). Regulation of Glucose Homeostasis by Glucocorticoids. Advances in Experimental Medicine and Biology.

[79] Pannacciulli N., Vettor R., Milan G., Granzotto M., Catucci A., Federspil G., De Giacomo P., Giorgino R., De Pergola G. (2003). Anorexia Nervosa Is Characterized by Increased Adiponectin Plasma Levels and Reduced Nonoxidative Glucose Metabolism. J. Clin. Endocrinol. Metab..

[80] Yamauchi T., Kamon J., Minokoshi Y., Ito Y., Waki H., Uchida S., Yamashita S., Noda M., Kita S., Ueki K. (2002). Adiponectin stimulates glucose utilization and fatty-acid oxidation by activating AMP-activated protein kinase. Nat. Med..

[81] Kadowaki T., Yamauchi T. (2005). Adiponectin and adiponectin receptors. Endocr. Rev..

[82] Luo Y., Liu M. (2016). Adiponectin: A versatile player of innate immunity. J. Mol. Cell Biol..

[83] Puchalska P., Crawford P.A. (2017). Multi-dimensional Roles of Ketone Bodies in Fuel Metabolism, Signaling, and Therapeutics. Cell Metab..

[84] Youm Y.-H., Nguyen K.Y., Grant R.W., Goldberg E.L., Bodogai M., Kim D., D’Agostino D., Planavsky N., Lupfer C., Kanneganti T.-D. (2015). The ketone metabolite β-hydroxybutyrate blocks NLRP3 inflammasome–mediated inflammatory disease. Nat. Med..

[85] Newman J.C., Verdin E. (2017). Beta-Hydroxybutyrate: A Signaling Metabolite. Annu Rev. Nutr..

[86] Evans M., Cogan K.E., Egan B. (2016). Metabolism of ketone bodies during exercise and training: Physiological basis for exogenous supplementation. J. Physiol..

[87] Mizushima N., Komatsu M. (2011). Autophagy: Renovation of Cells and Tissues. Cell.

[88] McLeod I.X., Jia W., He Y. (2012). The contribution of autophagy to lymphocyte survival and homeostasis. Immunol. Rev..

[89] Rocchi A., He C. (2017). Regulation of Exercise-Induced Autophagy in Skeletal Muscle. Curr. Pathobiol. Rep..

[90] Mooren F.C., Kruger K. (2015). Exercise, Autophagy, and Apoptosis. Prog. Mol. Biol. Transl. Sci..

[91] Palmowski J., Reichel T., Boßlau T.K., Krüger K. (2019). The effect of acute running and cycling exercise on T cell apoptosis in humans: A systematic review. Scand. J. Immunol..

[92] Mooren F.C., Krueger K., Ringseis R., Eder K., Liebisch G., Conrad K., Alack K., Maleki B.H. (2020). Combined effects of moderate exercise and short-term fasting on markers of immune function in healthy human subjects. Am. J. Physiol.-Regul. Integr. Comp. Physiol..

[93] Boada-Romero E., Martinez J., Heckmann B.L., Green D.R. (2020). The clearance of dead cells by efferocytosis. Nat. Rev. Mol. Cell Biol..

[94] Xu C., Mutwalli H., Haslam R., Keeler J.L., Treasure J., Himmerich H. (2024). C-reactive protein (CRP) levels in people with eating disorders: A systematic review and meta-analysis. J. Psychiatr. Res..

[95] Swenne I., Engström I. (2005). Medical assessment of adolescent girls with eating disorders: An evaluation of symptoms and signs of starvation. Acta Paediatr..

[96] Evans S.S., Repasky E.A., Fisher D.T. (2015). Fever and the thermal regulation of immunity: The immune system feels the heat. Nat. Rev. Immunol..

[97] Romanovsky A.A., Almeida M.C., Aronoff D.M., Ivanov A.I., Konsman J.P., Steiner A.A., Turek V.F. (2005). Fever and hypothermia in systemic inflammation: Recent discoveries and revisions. Front. Biosci..

[98] Wendt D., van Loon L.J.C., Lichtenbelt W.D.v.M. (2007). Thermoregulation during exercise in the heat: Strategies for maintaining health and performance. Sports Med..

[99] Gleeson M. (1998). Temperature Regulation During Exercise. Int. J. Sports Med..

[100] Keys A., Henschel A., Brožek J. (1950). The Biology of Human Starvation: Volume II.

[101] Luck P., Wakeling A. (1982). Set-Point Displacement for Behavioural Thermoregulation in Anorexia Nervosa. Clin. Sci..

[102] Kluger M.J., Ringler D.H., Anver M.R. (1975). Fever and survival. Science.

[103] Gutierrez E., Vazquez R., Beumont P.J. (2002). Do people with anorexia nervosa use sauna baths? A reconsideration of heat-treatment in anorexia nervosa. Eat. Behav..

[104] Gutiérrez E., Baysari M.T., Carrera O., Whitford T.J., Boakes R.A. (2006). High Ambient Temperature Reduces Rate of Body-Weight Loss Produced by Wheel Running. Q. J. Exp. Psychol..

[105] Gutierrez E., Cerrato M., Carrera O., Vazquez R. (2008). Heat reversal of activity-based anorexia: Implications for the treatment of anorexia nervosa. Int. J. Eat. Disord..

[106] Cerrato M., Carrera O., Vazquez R., Echevarría E., Gutierrez E. (2010). Heat makes a difference in activity-based anorexia: A translational approach to treatment development in anorexia nervosa. Int. J. Eat. Disord..

[107] Filteau S., Menzies R.A., Kaido T.J., O’Grady M.P., Gelderd J.B., Hall N.R. (1992). Effects of exercise on immune functions of undernourished mice. Life Sci..

[108] Hillebrand J., Derijke C., Brakkee J., Kas M., Adan R. (2005). Voluntary access to a warm plate reduces hyperactivity in activity-based anorexia. Physiol. Behav..

[109] Freyschuss U., Fohlin L., Thorén C. (1978). Limb circulation in anorexia nervosa. Acta Paediatr..

[110] Fohlin L., Freyschuss U., Bjarke B., Davies C.T.M., Thorén C. (1978). Function and dimensions of the circulatory system in anorexia nervosa. Acta Paediatr..

[111] Wenisch C., Narzt E., Sessler D.I., Parschalk B., Lenhardt R., Kurz A., Graninger W. (1996). Mild intraoperative hypothermia reduces production of reactive oxygen intermediates by polymorphonuclear leukocytes. Anesth. Analg..

[112] Romanovsky A., Székely M. (1998). Fever and hypothermia: Two adaptive thermoregulatory responses to systemic inflammation. Med. Hypotheses.

[113] Liu E., Lewis K., Al-Saffar H., Krall C.M., Singh A., Kulchitsky V.A., Corrigan J.J., Simons C.T., Petersen S.R., Musteata F.M. (2012). Naturally occurring hypothermia is more advantageous than fever in severe forms of lipopolysaccharide- and *Escherichia coli*-induced systemic inflammation. Am. J. Physiol.-Regul. Integr. Comp. Physiol..

[114] Kalm L.M., Semba R.D. (2005). They Starved So That Others Be Better Fed: Remembering Ancel Keys and the Minnesota Experiment. J. Nutr..

[115] Casper R.C. (2020). Might Starvation-Induced Adaptations in Muscle Mass, Muscle Morphology and Muscle Function Contribute to the Increased Urge for Movement and to Spontaneous Physical Activity in Anorexia Nervosa?. Nutrients.

[116] Dantzer R., Bluthé R.-M., Castanon N., Kelley K.W., Konsman J.-P., Laye S., Lestage J., Parnet P. (2007). Cytokines, Sickness Behavior, and Depression. Immunol. Allergy Clin..

[117] Nagata T., Tobitani W., Kiriike N., Iketani T., Yamagami S. (1999). Capacity to Produce Cytokines During Weight Restoration in Patients With Anorexia Nervosa. Psychosom. Med..

[118] Medzhitov R., Schneider D.S., Soares M.P. (2012). Disease Tolerance as a Defense Strategy. Science.

[119] Soares M.P., Teixeira L., Moita L.F. (2017). Disease tolerance and immunity in host protection against infection. Nat. Rev. Immunol..

[120] Birmingham C.L., Hodgson D.M., Fung J., Brown R., Wakefield A., Bartrop R., Beumont P. (2003). Reduced febrile response to bacterial infection in anorexia nervosa patients. Int. J. Eat. Disord..

[121] Rosales C. (2018). Neutrophil: A Cell with Many Roles in Inflammation or Several Cell Types?. Front. Physiol..

[122] Hutter G., Ganepola S., Hofmann W. (2009). The hematology of anorexia nervosa. Int. J. Eat. Disord..

[123] Gotch F.M., Spry C.J., Mowat A.G., Beeson P.B., Maclennan I.C. (1975). Reversible granulocyte killing defect in anorexia nervosa. Clin. Exp. Immunol..

[124] Sadiku P., Willson J.A., Ryan E.M., Sammut D., Coelho P., Watts E.R., Grecian R., Young J.M., Bewley M., Arienti S. (2021). Neutrophils Fuel Effective Immune Responses through Gluconeogenesis and Glycogenesis. Cell Metab..

[125] Suzuki K., Totsuka M., Nakaji S., Yamada M., Kudoh S., Liu Q., Sugawara K., Yamaya K., Sato K. (1999). Endurance exercise causes interaction among stress hormones, cytokines, neutrophil dynamics, and muscle damage. J. Appl. Physiol..

[126] Murray P.J., Wynn T.A. (2011). Protective and pathogenic functions of macrophage subsets. Nat. Rev. Immunol..

[127] Poon I.K.H., Lucas C.D., Rossi A.G., Ravichandran K.S. (2014). Apoptotic cell clearance: Basic biology and therapeutic potential. Nat. Rev. Immunol..

[128] Kawanishi N., Yano H., Yokogawa Y., Suzuki K. (2010). Exercise training inhibits inflammation in adipose tissue via both suppression of macrophage infiltration and acceleration of phenotypic switching from M1 to M2 macrophages in high-fat-diet-induced obese mice. Exerc. Immunol. Rev..

[129] Murray P.J., Allen J.E., Biswas S.K., Fisher E.A., Gilroy D.W., Goerdt S., Gordon S., Hamilton J.A., Ivashkiv L.B., Lawrence T. (2014). Macrophage activation and polarization: Nomenclature and experimental guidelines. Immunity.

[130] Banchereau J., Steinman R.M. (1998). Dendritic cells and the control of immunity. Nature.

[131] Wculek S.K., Khouili S.C., Priego E., Heras-Murillo I., Sancho D. (2019). Metabolic Control of Dendritic Cell Functions: Digesting Information. Front. Immunol..

[132] Collin M., McGovern N., Haniffa M. (2013). Human dendritic cell subsets. Immunology.

[133] O’Neill L.A.J., Pearce E.J. (2016). Immunometabolism governs dendritic cell and macrophage function. J. Exp. Med..

[134] Jain A., Pasare C. (2017). Innate Control of Adaptive Immunity: Beyond the Three-Signal Paradigm. J. Immunol..

[135] Smith-Garvin J.E., Koretzky G.A., Jordan M.S. (2009). T cell activation. Annu. Rev. Immunol..

